# Integrative analysis of gut microbiota, plasma metabolome, and gene expression identifies causal mediators in Graves’ disease pathogenesis

**DOI:** 10.3389/fmicb.2026.1704766

**Published:** 2026-08-03

**Authors:** Yuying Zhang, Jin’e Li, Yunfei Luo, Shuang Liu, Jianping Liu

**Affiliations:** 1 Department of Endocrinology and Metabolism, The Second Affiliated Hospital, Jiangxi Medical College, Nanchang University, Nanchang, Jiangxi, China; 2 Institute for the Study of Endocrinology and Metabolism in Jiangxi Province, Nanchang, Jiangxi, China; 3 Branch of National Clinical Research Center for Metabolic Diseases, Nanchang, Jiangxi, China; 4 Jiangxi Key Laboratory of Molecular Medicine, The Second Affiliated Hospital of Nanchang University, Nanchang, Jiangxi, China

**Keywords:** gene regulation, Graves’ disease, gut microbiota, immune inflammation, Mendelian randomization, plasma metabolites

## Abstract

**Background:**

Graves’ disease (GD) is characterized by hyperthyroidism and is influenced by genetic and environmental factors. The “gut-thyroid axis” establishes a connection between the gut microbiota and GD, yet the underlying potential mechanisms remain unclear. This study employed Mendelian randomization to investigate the causal relationships between the gut microbiota and GD, aiming to identify key microbial taxa and their metabolites, as well as to explore the regulatory roles of relevant genes in the pathogenesis of GD.

**Methods:**

We utilized the two-sample Mendelian randomization (MR) approach to evaluate the causal effects of gut microbiota and plasma metabolites on GD. Mediation analysis was conducted to explore the associations of metabolites in linking gut microbiota to GD. Additionally, we employed bioinformatics tools to identify GD-regulating genes within the gut microbiome and validated their expression levels in peripheral blood mononuclear cells from GD mouse models.

**Results:**

Mendelian randomization analysis identified eight gut microbes associated with GD, six of which were positively correlated with an increased risk, while two were negatively correlated. Additionally, 56 plasma metabolites exhibited potential causal relationships with GD; of these, 27 were positively associated with risk and 29 were negatively associated. Mediation analysis revealed that three bacteria influenced GD through five plasma metabolites. Specifically, the mannose to glycerol ratio and 1-(1-enyl-palmitoyl)-GPC (p-16:0) mediated the effect of *Dialister* on GD. Gamma-glutamylthreonine mediated the effect of *Oscillospira* on GD, whereas the ratios of acetylcarnitine (C2) to propionylcarnitine (C3) and adenosine 5′-diphosphate (ADP) to ornithine mediated the effect of MollicutesRF9 on GD. These microbiota regulate plasma metabolites, thereby affecting GD. TAGAP and HERC3 were significantly upregulated in the peripheral blood mononuclear cells of GD mice induced by recombinant adenovirus Ad-TSHR289, while NCEH1 and LYN were significantly downregulated, indicating their potential role in the regulation of GD.

**Conclusion:**

This study reveals that the composition of gut microbiota and its related metabolites promote the development of GD through the modulation of gene expression in peripheral blood mononuclear cells. Our findings have significant implications for the advancement of gut microbiota-based diagnostic techniques and targeted therapies for GD.

## Introduction

Graves’ disease (GD), also known as diffuse toxic goiter, is an organ-specific autoimmune disorder characterized by elevated thyroid hormone secretion. The global prevalence of GD is approximately 1.2%, with a notable gender disparity, exhibiting a female-to-male ratio of about 5:1, particularly among women aged 20–50 years (Smith and Hegedus, 2016; Kahaly, 2020; Liu et al., 2022). Typical manifestations of GD include hyperthyroidism, palpitations, and ocular disorders (Bartalena, 2013). The etiology of GD involves a complex interaction between susceptibility genes and environmental factors, including radiation, viral infections, medications, and levels of selenium and iodine (Vargas-Uricoechea, 2023). Currently, the main treatment methods include antithyroid drugs (ATDs), radioactive iodine (RAI), and thyroidectomy. However, the significant recurrence rate following ATDs treatment and the eventual onset of permanent hypothyroidism after RAI and thyroidectomy (Bartalena et al., 2022; Kahaly et al., 2018), underscore the necessity for innovative targeted prevention and treatment strategies.

The gut microbiota constitutes a complex and dynamic ecosystem that influences host nutrient metabolism and immune function, and is associated with various diseases (de Vos et al., 2022). Recent studies underscore gut microbial dysbiosis as a pivotal factor in the progression of autoimmune thyroid disease (Jiang et al., 2021; Liu et al., 2024; Cayres et al., 2021). The concept of the gut-thyroid axis has been employed to explore the relationship between the gut and thyroid, particularly concerning GD (Su et al., 2020; Ishaq et al., 2022; Su et al., 2020). Gut microbiota are considered to be tightly linked to thyroid function status and the severity of GD (Chen et al., 2021; Biscarini et al., 2023). Epidemiological studies examining the association between gut microbiota and GD have yielded conflicting findings. For instance, some reports indicate that the abundances of *Faecalibacterium*, *Bacteroides*, *Blautia*, and *Ruminococcus* in the feces of GD patients are decreased compared to those in control groups (Biscarini et al., 2023; Chang et al., 2021), while other studies have reported increased abundances of these bacteria in the feces of GD patients compared to controls (Jiang et al., 2021; Liu et al., 2024; Chen et al., 2021).

In patients with GD, the abundance of *Lactobacillus* shows a positive correlation with thyroid-stimulating hormone receptor antibodies (TRAb), thyroperoxidase antibodies (TPOAb), and thyroglobulin antibodies (TgAb) (Jiang et al., 2021; Chen et al., 2021). Conversely, *Synergistetes* exhibits a negative correlation with thyroid-stimulating antibodies (TSAb). Additionally, *Ruminococcus*, *Bifidobacterium*, and *Veillonella* are positively correlated with TRAb (Cornejo-Pareja et al., 2020; Yang et al., 2022), while other bacterial groups, such as *Prevotella 9*, *Actinomyces odontolyticus*, and *Actinomyces negativus*, positively correlate with TPOAb (Chang et al., 2021). Despite these findings, the causal link and underlying mechanisms between gut microbiota and GD remain unclear.

Gut microbiota produce a variety of bioactive metabolites through the metabolism of food and other substances. These metabolites significantly impact host health and play a crucial role in the development and progression of GD. Research indicates that GD is associated with metabolic disorders, characterized by decreased plasma levels of short-chain fatty acids (SCFAs) in affected patients. This reduction exacerbates intestinal barrier damage and elevates serum lipopolysaccharide (LPS) levels (Su et al., 2020). Furthermore, propionic acid supplementation in GD mouse models has been shown to enhance immune function in GD patients by inhibiting lymphocyte histone deacetylase (HDAC) activity, which leads to an increase in regulatory T (Treg) cells and a decrease in Th17 cells.

Mendelian randomization (MR) is an analytical method that employs genetic variants as instruments to simulate the conditions of randomized controlled trials (RCTs), thereby facilitating the determination of causal relationships between risk factors and diseases. This approach reduces the impact of confounding variables and addresses the issue of reverse causality, thus improving the precision of causal inference (Zabor et al., 2020; Birney, 2022).

This study employs MR to explore the causal relationship between gut microbiota and its metabolites in the context of GD. We utilized comprehensive data from large-scale GWASs of GD patients, alongside gut microbial profiles and plasma metabolome sequencing. Furthermore, we conducted bioinformatics analyses to identify genes associated with the regulation of GD-related target microbial communities, and we validated these findings using a GD mouse model. This approach investigates the pathogenesis of GD through the gut microbiome, offering novel insights and potential strategies for therapeutic intervention and prevention.

## Methods

### Study design and the assumption of MR

**Figure 1:**
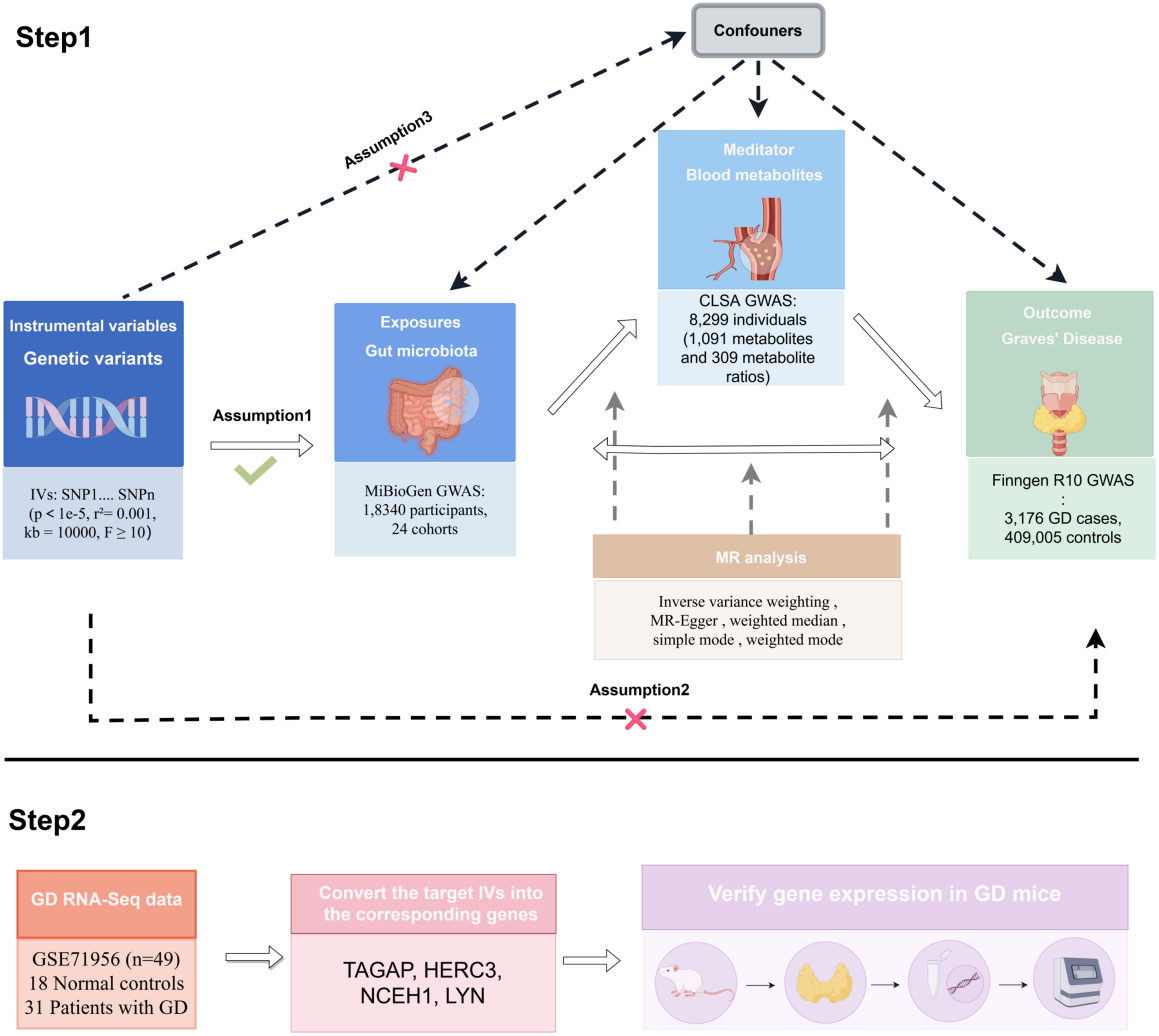
Overview of research design. Step 1: Conduct a Mendelian randomization analysis to explore the causal links between gut microbiota, plasma metabolites, and GD, as well as to identify metabolites that may mediate the association between microbiota and GD. Step 2: Analyze the Gene Expression Omnibus (GEO) dataset GSE71956 to identify genes associated with the development of GD. The genes linked to the gut microbiota IVs identified in this study were compared with those from the GEO database. This overlap analysis revealed a set of genes implicated in the pathogenesis of GD, the expression of which was validated in mice using Q-PCR.

Figure 1 illustrates the design process of this study. Genetic variants associated with gut microbiota derived from GWAS summary results were used as instrumental variables to conduct univariate MR analysis to determine the causal relationship between gut microbiota and GD. Then, reverse MR analysis was performed by setting GD as the exposure and the abundance of the selected group as the result. Next, a two-step MR method was used to study the causal relationship between gut microbiota and plasma metabolites, and to evaluate the effects of these metabolites on GD (Sanderson, 2021). In Mendelian randomization studies, instrumental variables (IVs) must satisfy three critical assumptions to ensure the validity of causal estimates (Larsson et al., 2023): (1) single nucleotide polymorphisms (SNPs) should exhibit a strong correlation with the exposure (gut microbiota/plasma metabolites) and achieve genome-wide significance; (2) the selected IVs must be independent of any confounders that could affect the exposure-outcome (gut microbiota/plasma metabolites-GD) relationship; and (3) in the main analysis of exposure (gut microbiota/plasma metabolites) and outcome (GD), genetic instrumental variables should only affect outcome (GD) through corresponding exposure (gut microbiota/plasma metabolites), excluding other independent pathways; in the mediation analysis, the relevant instrumental variables also need to ensure that the impact of exposure (gut microbiota) on outcome (GD) is completely mediated by specific metabolites. Consequently, the SNPs selected in this study satisfy these criteria: they exhibit strong exposure associations, are subject to random genetic assignment, remain unconfounded by measured/unmeasured covariates, and operate exclusively via exposure-mediated mechanisms to affect GD outcomes.

### Data sources

This study utilized GWAS data on gut microbiota from the MiBioGen Consortium, which genotyped 18,340 participants from 24 cohorts. It examined the V4, V3–V4, and V1–V2 variable regions of the 16S rRNA gene in fecal microbiota to analyze microbial composition through direct taxonomic classification (Kurilshikov et al., 2021). The GWAS dataset of gut microbiota encompasses 211 taxa, including 131 genera, 35 families, 20 orders, 16 classes, and 9 phyla, with 12 classified as unknown genera. Consequently, 196 taxonomic units were utilized as taxonomic units for analysis in this study. Furthermore, GWAS data on the plasma metabolome were obtained from the Canadian Longitudinal Study of Aging (CLSA) cohort, which comprises 8,299 individuals and includes 1,091 blood metabolites and 309 metabolite ratios (Chen et al., 2023).

The GWAS summary data for GD were obtained from the Finngen R10 consortium, which consists of 3,176 cases and 409,005 controls diagnosed according to the International Classification of Diseases (Kurki et al., 2023). For further details regarding each GWAS data source, please refer to the cited papers. When the selected instrumental variables are sufficiently robust, the influence of overlapping individuals on statistical power is minimal (Zhu et al., 2018). This research involves a secondary analysis of publicly accessible GWAS summary data. Ethical approval was secured for each initial GWAS study, and since this study did not utilize individual-level data, no new ethics review board approval is necessary.

### Selection criteria of instrument variables

Instrumental variables were selected based on SNPs significantly associated with exposure factors (gut microbiota or metabolites) from the GWAS database. Due to the small sample size of gut microbiome and metabolites GWAS, in order to obtain sufficient instrumental variables, this study used a loose threshold of *p* < 1e-5 and corrected the potential bias through sensitivity analysis (Sanna et al., 2019; Liu et al., 2022). To ensure the independence of the instrumental variables and to avoid collinearity issues, SNPs in linkage disequilibrium (LD) were removed by setting *r*^2^ = 0.001 within a window of 10,000 kb. The *F* value is used to measure the strength of the correlation between the genetic instrumental variables and the exposure factors; SE is the standard error corresponding to the *β* coefficient, which indicates the degree of correlation between carrying an allele and the change of exposure factor level (Bowden et al., 2016; Wang et al., 2022). The F-statistic, calculated as *F* = *β*^2^/SE^2^, was used to select SNPs with values exceeding 10 to ensure data quality (Pierce et al., 2011).

### Bayesian weighted Mendelian randomization verification

The Bayesian weighted Mendelian randomization (BWMR) method effectively enables the investigation of causal relationships between exposure and outcomes, while also evaluating the impact of uncertainty arising from weak effect and weak level pleiotropy through standard error and *p*-value analysis based on GWAS summary statistics (Wang et al., 2022). Therefore, it is imperative to further validate the findings obtained from MR Analysis. Therefore, we employ BWMR to further validate the reliability of IVW (*p* < 0.05). To improve the accuracy of the results, we performed a Robust Bayesian analysis of the MR results using the RBMR package.^1^

### Development of a mouse model for Graves’ disease and assessment of thyroid function along with associated antibodies

The recombinant adenovirus containing amino acids 1–289 of the thyroid-stimulating hormone receptor (TSHR) (Ad-TSHR289) was purchased from Bioengineering (Shanghai) Co., Ltd. All animal experiments adhered to the Animal Research: Reporting of *In Vivo* Experiments guidelines and complied with the UK Animals (Scientific Procedures) Act 1986, the European Union Directive 2010/63/EU on animal experimentation, or the National Research Council’s Guide for the Care and Use of Laboratory Animals. At the conclusion of the study, all animals were euthanized using CO_2_ anesthesia. The experiment involved 6–8 week-old specific pathogen-free (SPF) female BALB/c mice, sourced from Jiangxi Kress Biotechnology Co., Ltd., and maintained in a standardized SPF environment. The research adhered to the ethical standards outlined in the Guide for the Care and Use of Laboratory Animals, with all experimental procedures reviewed and approved by the Animal Experiment Ethics Committee at Nanchang University (approval number: NCULAE-20221031043). Following a 7-day acclimatization period, mice were immunized to establish the model through the injection of recombinant adenovirus Ad-TSHR289 into the quadriceps muscle, except for the control group which retained 6 mice. The viral suspension was prepared in 25 μL of sterile diluent, with a single injection dose of 1 × 10^9^ virus particles (VP), administered intramuscularly using a microsyringe. The immunization protocol consisted of three administrations, delivered at 21-day intervals, to establish a stable animal model. Three weeks after the final immunization, blood was collected via the tail vein for subsequent analysis.

### Examination of thyroid function and thyroid-related autoantibody levels

Serum levels of free triiodothyronine (FT3), free thyroxine (FT4), and TRAb in mice were assessed using enzyme-linked immunosorbent assay (ELISA) kits from Jianglai Biotechnology (Shanghai, China) and analyzed with a SpectraMax microplate reader (Molecular Devices, Sunnyvale, CA, United States), in accordance with the manufacturers’ guidelines. The normal range was defined as the mean plus or minus the standard deviations (SD) of the control group mice.

### qRT-PCR

RNA was isolated from PBMCs utilizing TRIzol Reagent (TIANGEN, DP405-02). The concentration and purity of RNA were assessed using a NanoDrop ND-2000 spectrophotometer (Thermo Fisher Scientific). Complementary DNA (cDNA) was synthesized from RNA using the TaKaRa Reverse Transcription Kit (RR047B). Gene expression was evaluated using SYBR Green real-time PCR (Bio-Rad), with β-actin serving as the normalization reference. Specific primers for β-actin, *TAGAP*, *HERC3*, *NCEH1*, and *LYN* were used (primer sequences are provided in Supplementary Table S1). The comparative cycle threshold (2^−ΔΔCt^) method was employed to analyze quantitative reverse transcription polymerase chain reaction (RT-qPCR) data. Primers were synthesized by Sangon Biotech (Shanghai) Co., Ltd., China.

### Statistical analyses

This study utilized univariate MR analysis to examine the causal effects of gut microbiome and plasma metabolites on GD. We utilized five methods to evaluate causality: inverse variance weighting (IVW), the MR-Egger method, the weighted median method, the simple mode method, and the weighted mode method, with inverse variance weighting serving as the primary approach. Heterogeneity in MR refers to the problem that the same SNPs from different studies can have different estimated effects and *p*-values for the associated exposure/outcome. To detect the degree of heterogeneity, Cochrane’s *Q* test was employed (Burgess and Thompson, 2017). Q_Pval > 0.05 indicates the absence of significant heterogeneity among the estimates from each SNP. MR-Egger regression were employed to examine the possible horizontal pleiotropy effect (*p* < 0.05 was judged significant) (Burgess and Thompson, 2017). Among these five methods, only causal effects with a *p*-value less than 0.05 were considered significant. Additionally, these methods were also applied to evaluate the reverse causal relationship between gut microbiota and GD. We conducted an enrichment analysis of the identified plasma metabolites using MetaboAnalyst 5.0.^2^

We utilized a two-step Mendelian randomization (TSMR) approach to explore whether plasma metabolites mediate the association between gut microbiota and GD (Sanderson, 2021). Building on the established causal effect of gut microbiota on GD, we analyzed the causal effect of mediators (plasma metabolites) on GD and the causal effect of exposure (gut microbiota) on the mediators. When evidence indicates that gut microbiota influences plasma metabolites, which in turn affect GD, we apply the coefficient product method to assess the mediating effect, defined as β1*β2. The direct effect is calculated as the total effect minus the mediating effect, and the mediation ratio is determined using the formula ρM = β1*β2/β0 (Carter et al., 2021; Chen et al., 2022). Where ρM is the proportion of mediation effect of the mediator plasma metabolites, β1 is the MR causal effect of exposure (gut microbiota) on mediator (plasma metabolites), β2 is the MR causal effect of mediator (plasma metabolites) on outcome (GD), β1*β2 represents the ‘indirect ‘effect through mediation (plasma metabolites), and β0 is the ‘total ‘effect of exposure (gut microbiota) on outcome (GD).

Statistical analyses were conducted using R software version 4.3.1, employing the TwoSample MR (v0.5.6) and MR-Presso (v1.0) packages for MR analysis.

GraphPad Prism 10 was used to analyze the experimental data, which are presented as mean ± standard deviation. A *t*-test was conducted to analyze the differences between the two groups, with a *p*-value of less than 0.05 considered statistically significant.

## Results

### Causal effects of gut microbiota on GD

**Figure 2:**
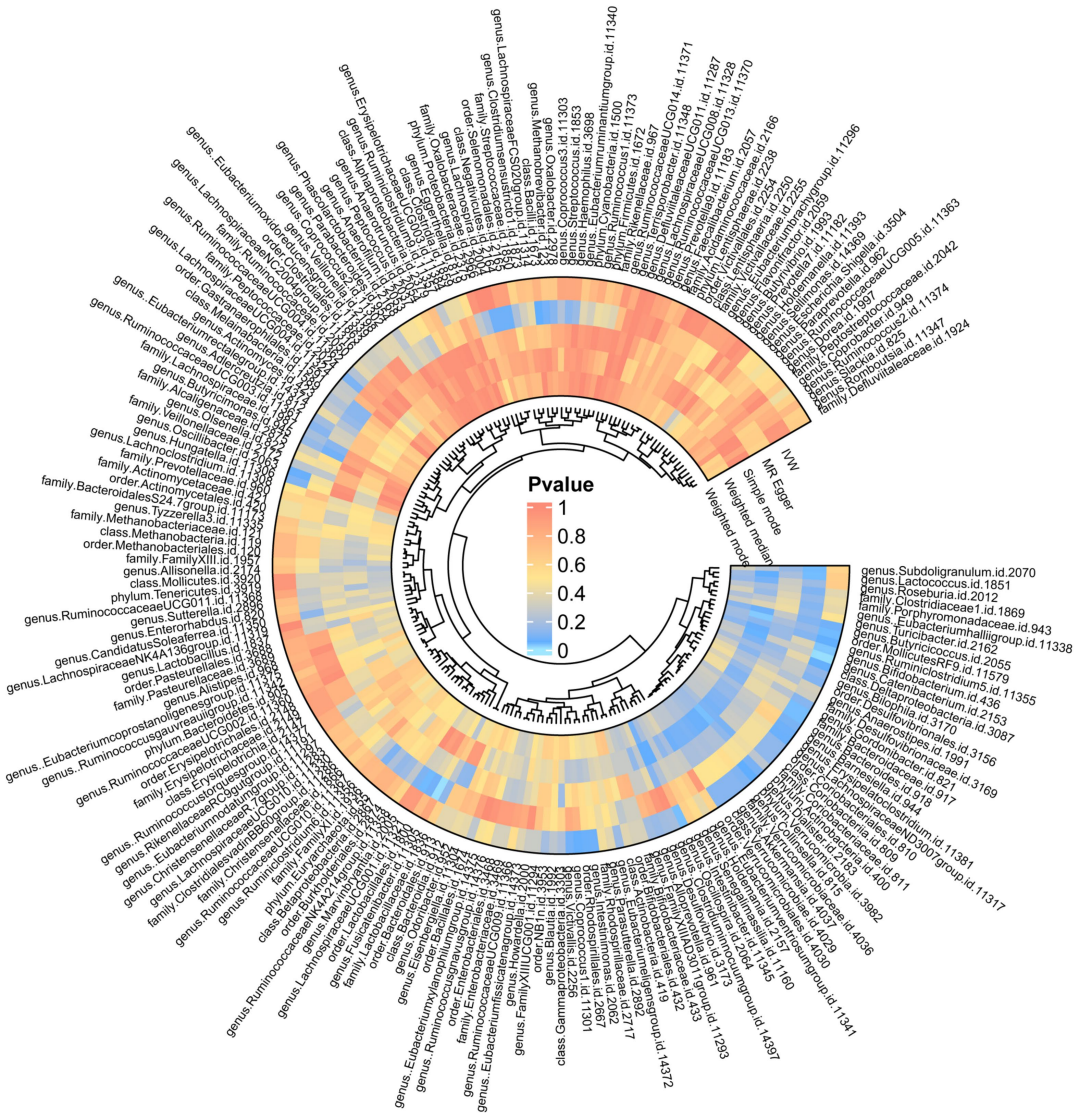
Circular heat map showing the causal effect of gut microbiota on GD risk. A causal link between 196 intestinal microbiota groups and GD was identified using five MR analysis methods (*p* < 0.05). The figure sequentially presents the names of intestinal microbiota taxa and the *p* values derived from IVW, MR-Egger, simple mode, weighted median, and weighted mode analyses, arranged from the outermost to the innermost layer.

**Figure 3:**
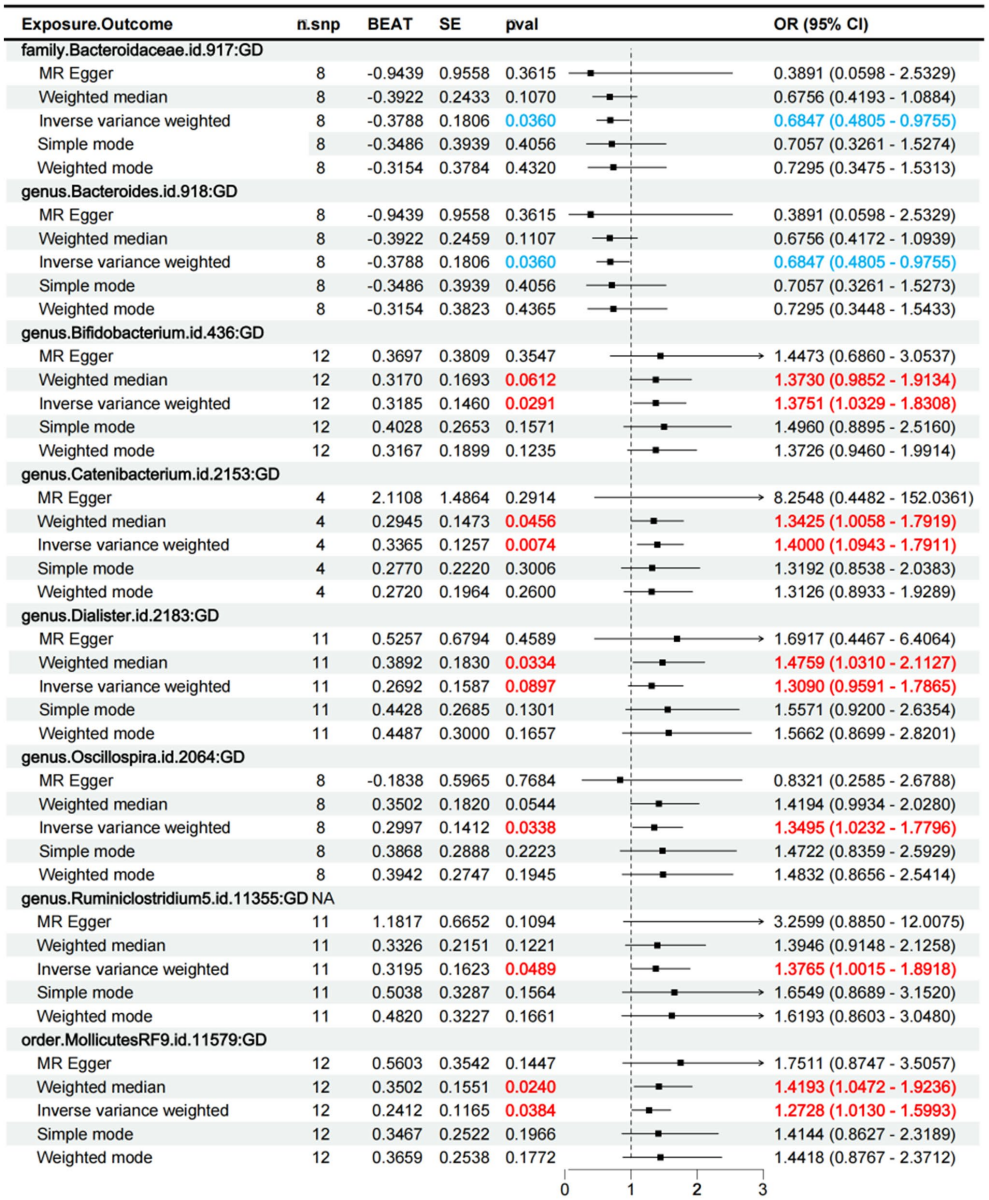
Forest plot of the potential causal relationship between gut microbiota and GD. CI stands for confidence interval, and SE represents standard error.

This study employed five two-sample MR methods—IVW, MR Egger, weighted median, simple mode, and weighted mode—to examine the causal link between the relative abundance of gut microbiota and GD across five taxonomic levels: phylum, class, order, family, and genus, as illustrated in Figure 2. In the forward MR analysis of gut microbiota and GD, as shown in Supplementary Figure S2, the order MollicutesRF9, family Bacteroidaceae, and the genera *Bifidobacterium*, *Catenibacterium*, *Dialister*, *Oscillospira*, *Ruminiclostridium5*, and *Bacteroides* were linked with GD. The impact of various SNPs on GD is depicted in Supplementary Table S2 and Supplementary Figure S1. As shown in Figure 3, the order MollicutesRF9 and genera *Bifidobacterium*, *Catenibacterium*, *Dialister*, *Oscillospira*, and *Ruminiclostridium5* were positively associated with GD, suggesting that these groups may serve as risk factors contributing to the progression of the condition. Conversely, the family Bacteroidaceae and genera *Bacteroides* were negatively associated with GD, indicating that these groups may play a protective role in inhibiting disease progression. We further validated eight significantly related bacterial taxa with BWMR (Supplementary Table S3). The results showed that the GD-related family Bacteroidaceae, genera *Bacteroides* and *Ruminiclostridium5* did not pass the BWMR test (*p* > 0.05), while the remaining five bacterial taxa continued to show robust statistical significance.

We conducted heterogeneity, pleiotropy, and leave-one-out analyses to evaluate the reliability, stability, and potential biases of the MR results, as well as the influence of instrumental variables. Cochran’s *Q* test indicated no significant heterogeneity among the eight taxa in both MR-Egger and IVW analyses (refer to Supplementary Table S4 and Supplementary Figure S3). The pleiotropy analysis indicated that the eight microbial taxa did not exhibit horizontal pleiotropy in their effects on GD (Supplementary Table S5). For bacteria with a *p*-value < 0.05 in the IVW analysis that also passed the heterogeneity and pleiotropy tests, a leave-one-out analysis was conducted. This involved sequentially excluding each relevant SNP and recalculating the combined effects of the remaining SNPs (Supplementary Figure S4). After the exclusion of each SNP, the total error bar remained relatively unchanged, with all error bars consistently falling to the right or left of zero.

### Causal effect of GD on gut microbiota

**Figure 4:**
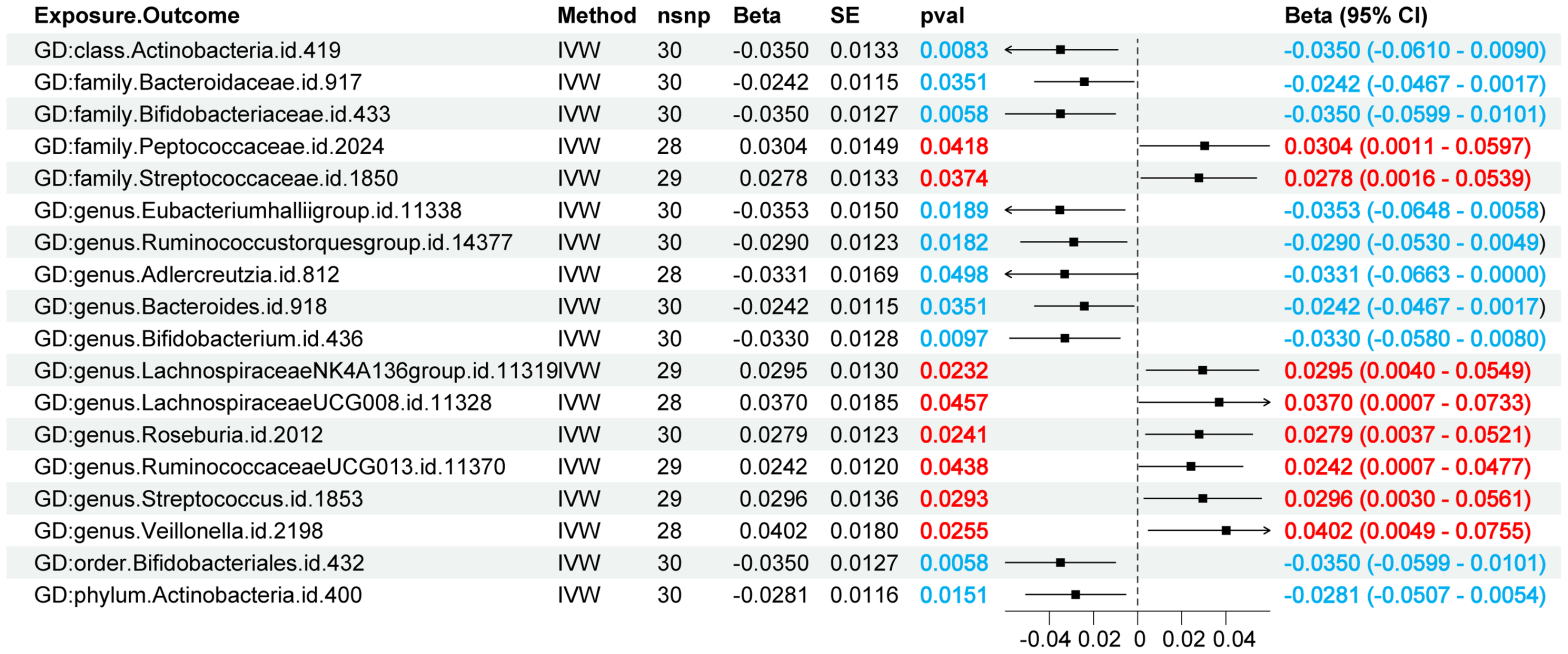
Forest plot to visualize the causal effects of GD to GM. CI, confidence interval; SE, standard error.

We performed a reverse MR analysis to investigate the causal relationship between gut microbiota and GD, aiming to identify potential confounding factors and validate their association (Figure 4). Our findings indicate that GD does not exert a causal impact on the abundance of specific gut microbial taxa, including the genera *Catenibacterium*, *Dialister*, *Oscillospira*, *Ruminiclostridium5*, and the order MollicutesRF9. Simultaneously, our analysis identified positive associations between GD and eight gut microbiota features, such as the families Peptococcaceae and Streptococcaceae, as well as the genus *Lachnospiraceae* NK4A136 group, suggesting that GD may facilitate the proliferation of these microorganisms. Conversely, GD exhibited negative correlations with 10 gut microbiota features, including the phylum Actinobacteria, the class Actinobacteria, the order Bifidobacteriales, the family Bifidobacteriaceae, and the genus *Bifidobacterium*, indicating that the progression of GD may inhibit the growth of these bacteria.

Heterogeneity analysis indicated no statistically significant differences (*p* > 0.05) across all data, except for the genus *Eubacterium hallii* group (Supplementary Table S6). Pleiotropy analysis suggested that the causal effects of GD on the *E. hallii* group and Veillonella could not exclude horizontal pleiotropy (Supplementary Table S7). Collectively, these results demonstrate a unidirectional causal relationship between GD and the genera *Catenibacterium*, *Dialister*, *Oscillospira*, *Ruminiclostridium5*, and the order MollicutesRF9, which stems from the influence of gut microbiota on GD.

### Causal relationships between plasma metabolites and GD

**Figure 5:**
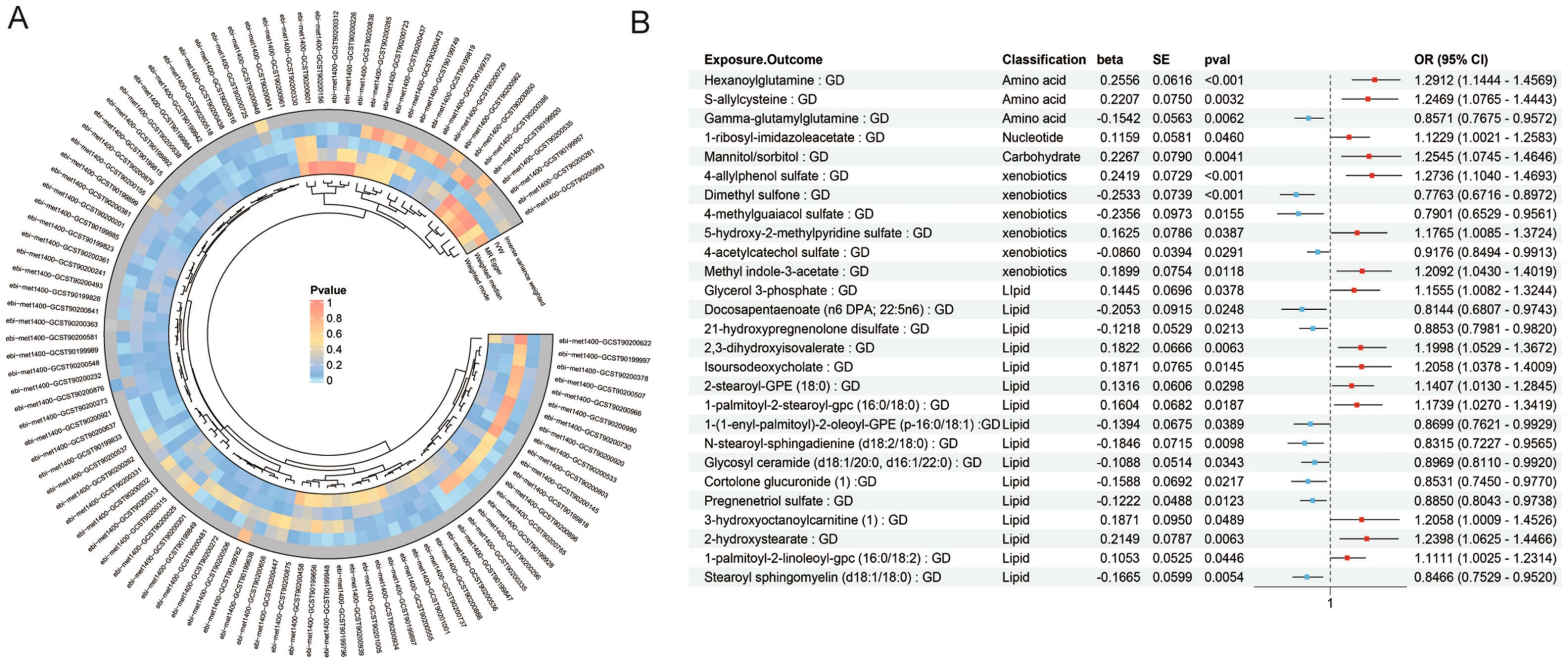
Causal effects of the plasma metabolites on GD. **(A)** Screening of the causal effects of the plasma metabolites on GD. **(B)** Forest plot of the potential causal relationship between plasma metabolites and GD.

Figure 5A and Supplementary Table S8 highlight 56 metabolites linked to GD, identified via the IVW method, with consistent directionality across five methods and no signs of heterogeneity or pleiotropy (Supplementary Tables S9, S10). Among the 56 metabolites, 27 are recognized compounds, including amino acids, carbohydrates, nucleotides, xenobiotics, and lipids. Figure 5B illustrates the causal relationships between GD and three amino acids, one carbohydrate, one nucleotide, and six exogenous drugs, revealing seven positive and four negative causal relationships with GD. Furthermore, 16 lipids were found to have causal effects on GD, with eight exhibiting positive effects and eight showing negative effects (Figure 5B).

**Figure 6:**
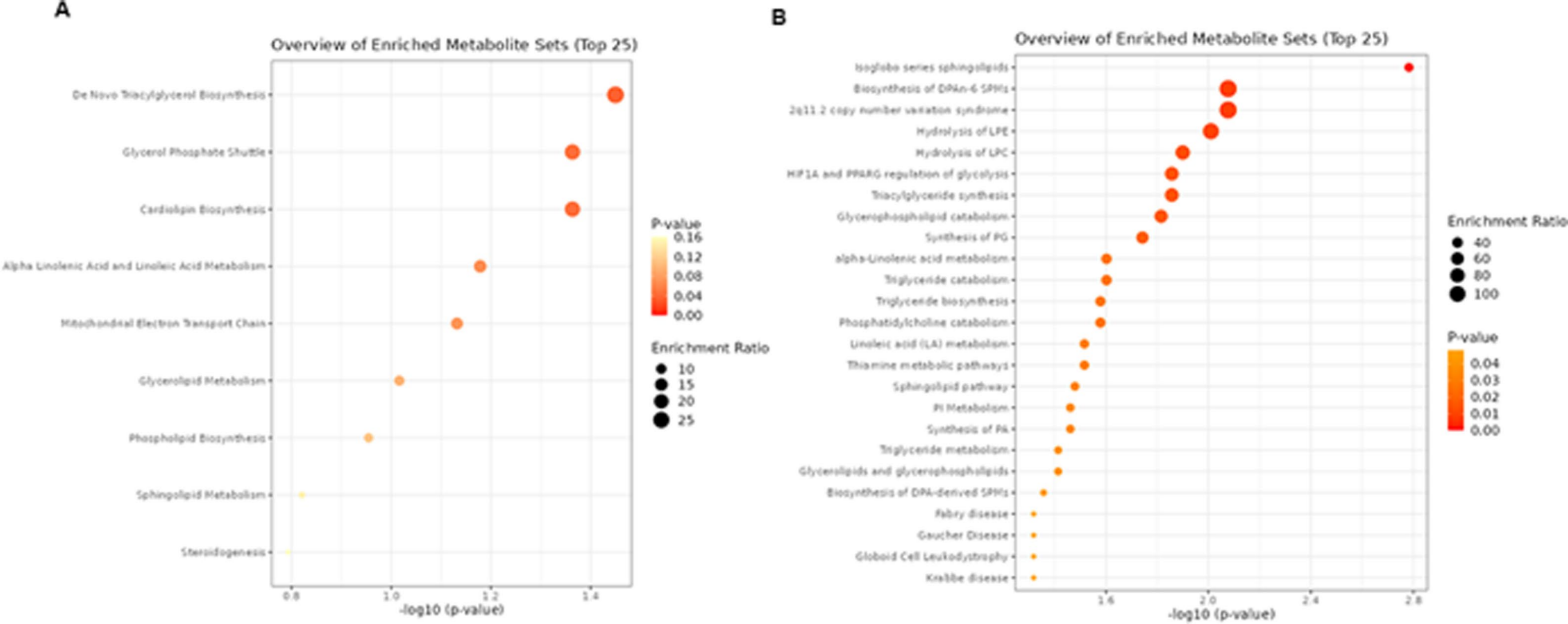
Enrichment analysis findings on how plasma metabolites causally affect GD. **(A)** Enrichment analysis of the causal impact of plasma metabolites on GD was conducted using the Small Molecule Pathway Database (SMPDB). **(B)** Enrichment analysis of the causal impact of plasma metabolites on GD was conducted using the Drug-related Metabolism Database.

The enrichment analysis of the identified metabolites revealed significant enrichment in pathways, including *De Novo* Triacylglycerol Biosynthesis, Glycerol Phosphate Shuttle, Cardiolipin Biosynthesis, Isoglobo series sphingolipids, and DPAn-6 SPM Biosynthesis (Figure 6).

**Figure 7:**
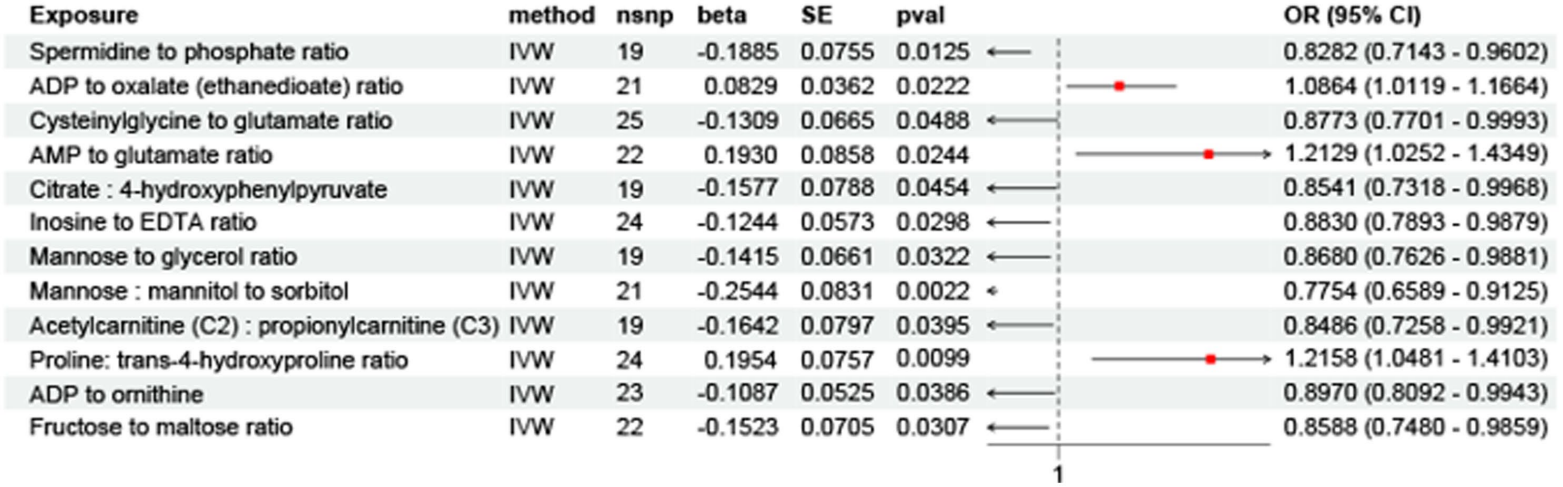
Illustrates the causal impact of plasma metabolite ratios on GD.

In addition, 12 metabolite ratios (Figure 7) and 14 unknown metabolites were associated with GD. Although the effects observed were only nominally significant, none reached statistical significance. Furthermore, no heterogeneity or pleiotropy was detected in any of the aforementioned associations (Supplementary Tables S9, S10).

### Mediation analysis of plasma metabolites

**Figure 8:**
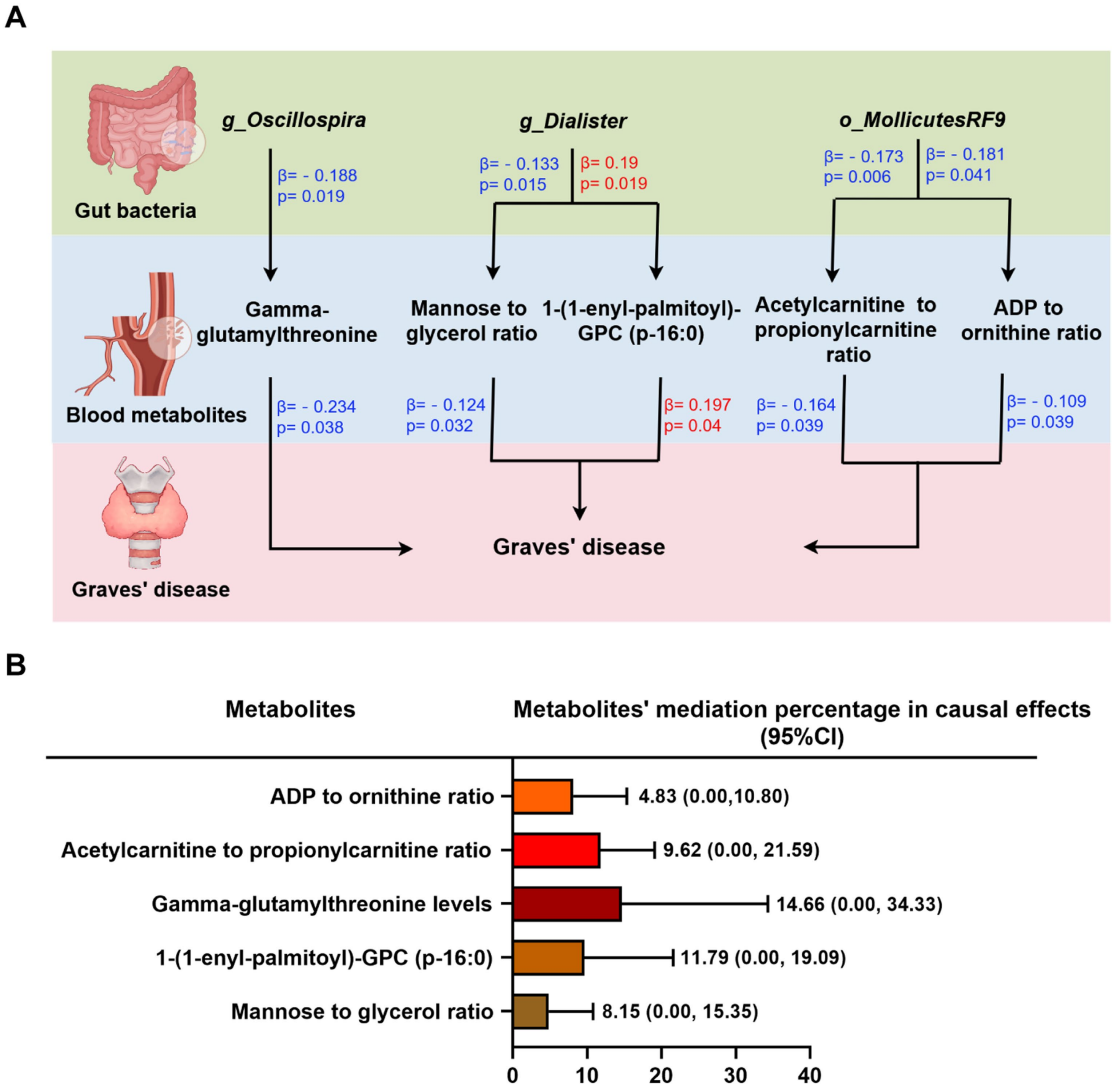
Plasma metabolites mediate the causal relationship between gut microbiota and Graves’ disease. **(A)** The mediation analysis explores the pathway ‘GM – blood metabolites – Graves’ disease. Red-β indicated positive effects, whereas blue-β represented negative effects. **(B)** The mediation role of plasma metabolites in the influence of gut microbiota on Graves’ disease.

Based on the identified gut microbiota and plasma metabolites, a two-step Mendelian randomization method was used to explore the mediating role of plasma metabolites in the effect of gut microbiota on GD. Supplementary Table S11 presents the effects of various SNP metabolites, while Supplementary Table S12 displays the MR analysis results concerning the impact of gut microbiota on metabolites. Figure 8A illustrates the identification of five mediation pathways. Gamma-glutamylthreonine was found to mediate 14.66% (95% CI = 0.00% − 34.33%, Figure 8B) of the effect of Oscillospira on GD. The mannose to glycerol ratio mediated 4.83% (95% CI = 0.00% − 10.80%, Figure 8B) of the effect of Dialister on GD. Furthermore, 1-(1-enyl-palmitoyl)-GPC (p-16:0) accounted for 9.62% (95% CI: 0.00% − 21.59%, Figure 8B) of Dialister’s impact on GD. The ratio of acetylcarnitine (C2) to propionylcarnitine (C3) contributed 11.79% (95% CI = 0.00% − 19.09%, Figure 8B) to the impact of MollicutesRF9 on GD. Additionally, the influence of MollicutesRF9 on GD was mediated by the adenosine 5′-diphosphate (ADP) to ornithine ratio, accounting for 8.15% (95% CI = 0.00% − 15.35%, Figure 8B).

### Genetic analysis

**Figure 9:**
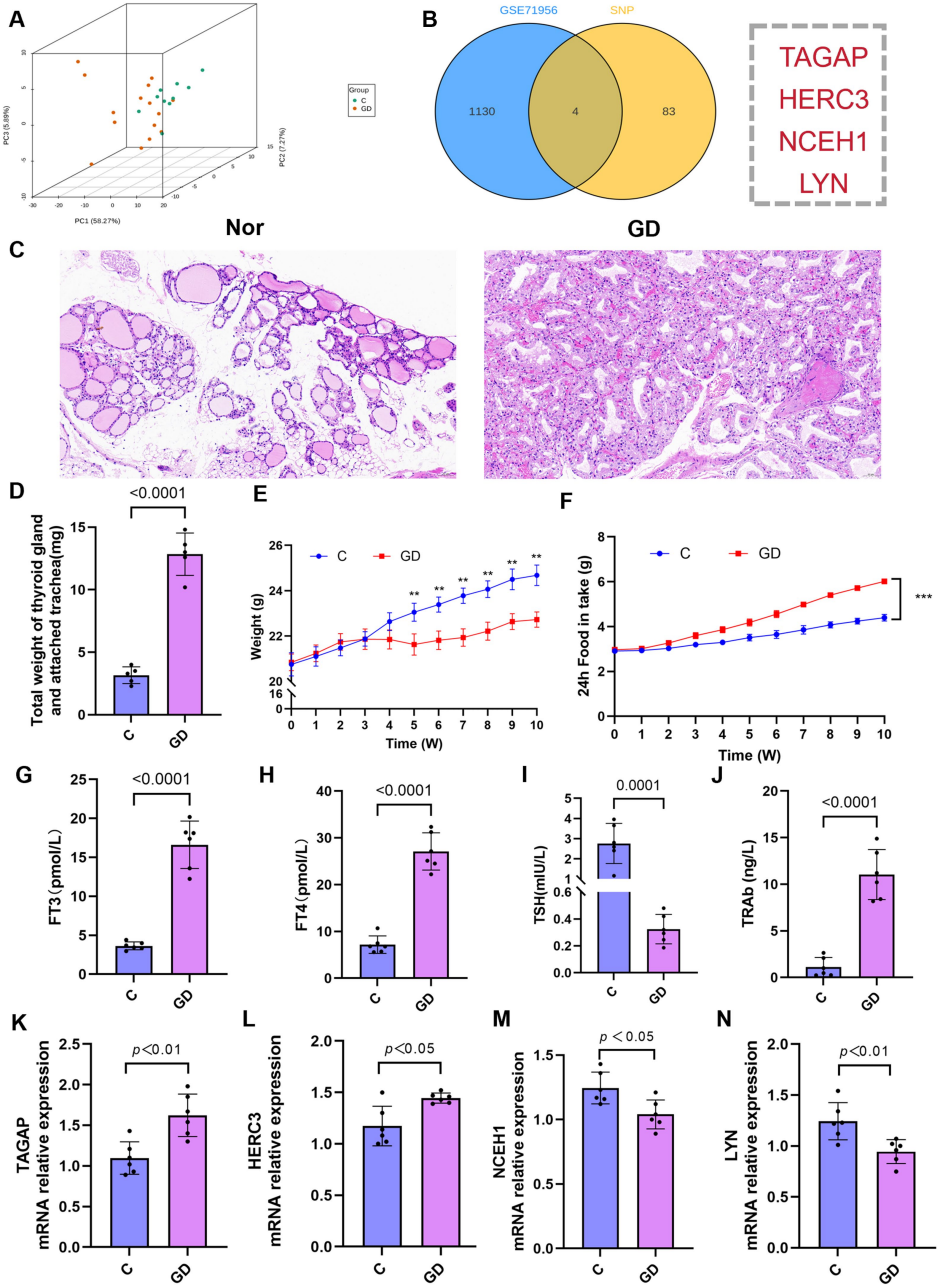
Potential mediator genes involved in the regulation of GD by gut microbiota. **(A)** PCA analysis of the GD-related gene expression comprehensive gene database (GSE71956). **(B)** Venn diagram of GD-related genes from the GEO database and the corresponding genes of the IVs of the three GD-related intestinal microbiota identified in this study. **(C)** HE staining of thyroid tissues of control mice and GD model mice. **(D)** Statistical graph of total thyroid weight of mice. **(E)** Curve of body weight over time for each mouse in each group. **(F)** Curve of food intake over time for each mouse in each group. **(G–J)** Statistical graphs of FT3, FT4, TSH, and TRAb in mouse serum. **(K–N)** qRT-PCR results of gene expression levels in mouse peripheral blood mononuclear cells. An exact 2-sample *t*-test was used for statistical analysis. Data are presented as mean ± SEM (*n* = 6). **p < 0.01, ***p < 0.001 vs. Control group by two-tailed independent samples *t*-test.

We also performed an extensive analysis of genes potentially affected by the gut microbiota, complementing the metabolite profile study. Initially, we examined the GEO dataset GSE71956 to identify genes associated with the progression of GD (Figure 9A).

Genes linked to the 12 GD-associated gut microbiota IVs identified in this study were cross-referenced with those in the GEO database, revealing GD pathogenesis-related genes: *TAGAP*, *HERC3*, *NCEH1*, and *LYN* (Figure 9B). To validate these findings, we created a GD mouse model using recombinant adenovirus Ad-TSHR289 to assess the expression of these genes. Figures 9C–J demonstrate that the GD group exhibited significantly higher serum levels of FT3, FT4, and TRAb compared to the control group (*p* < 0.01), whereas TSH levels were significantly lower (*p* < 0.0001). These findings validate the successful implementation of the GD model. Primers were designed for the genes *TAGAP*, *HERC3*, *NCEH1*, and *LYN* to quantify their expression in PBMCs from both GD and control mice. Figures 9K–N illustrate that under GD conditions, *TAGAP* and *HERC3* expression levels were significantly increased (*p* < 0.01), whereas *NCEH1* and *LYN* expression levels were significantly decreased compared to the control group. These findings suggest that gut microbiota may influence GD through specific genetic pathways.

## Discussion

Although the intestine and thyroid belong to different systems and are located at a considerable distance from each other, they can interact in both health and disease states (Jiang et al., 2022; Bowden et al., 2016). The gut-thyroid axis provides new directions for the treatment strategies of thyroid diseases. Previous studies have linked gut dysbiosis with thyroid cancer, Hashimoto’s thyroiditis, and other thyroid diseases (Jiang et al., 2022). This study reveals some new perspectives through MR analysis methods and animal experiments. Firstly, we established a causal relationship between the onset of GD and eight gut microbial taxa, including *Catenibacterium*, *Dialister*, *Oscillospira*, *Ruminiclostridium5*, and MollicutesRF9. Secondly, we demonstrated that genes such as *TAGAP*, *HERC3*, *NCEH1*, and *LYN* may participate in the regulation of GD through the gut microbiota-mediated gut-thyroid axis. Finally, our research indicates that three microbial taxa indirectly influence GD by affecting five specific metabolites.

With the rapid development of gut microbiome metagenomics and metabolomics, our understanding of the complex interactions between the microbiome and GD is gradually unfolding. Disruption of the gut microbiome composition and increased intestinal permeability may allow bacteria and harmful metabolites to enter the bloodstream, collectively leading to inflammation infiltration in blood and thyroid tissues, as well as immune imbalance, which are key factors contributing to GD (Zheng et al., 2021). Our study identified eight microbial taxa that may be involved in the development of GD, among which *Bifidobacterium*, *Catenibacterium*, *Dialister*, *Oscillospira*, *Ruminiclostridium5*, and MollicutesRF9 may increase the risk of GD, while Bacteroidaceae family and *Bacteroides* genus appear to have a protective effect. These findings are consistent with previous gut microbiome sequencing results conducted in GD patients (Ishaq et al., 2018). However, it has been reported that Bifidobacterium is found at low abundance in GD patients while the abundance of Bacteroides is increased (Jiang et al., 2021; Ishaq et al., 2018). In addition, Yang et al. demonstrated a decrease in the abundance of Dialister and Oscillospira in patients with GD, with a rebound in Dialister levels following antithyroid drug treatment (Yang et al., 2022). These differences may be attributed to variations in sample characteristics, genetic profiles, measurement methods, and statistical analysis techniques, particularly regarding the selection of SNPs utilized in our study. Notably, our research also revealed an association between the genera *Catenibacterium*, *Ruminiclostridium5*, and MollicutesRF9 and GD, a connection that has not been previously reported. Furthermore, in the reverse Mendelian randomization analysis of gut microbiota and GD, GD exhibited a positive influence on the family Bacteroidaceae and the genus *Bifidobacterium*, indicating the presence of a negative feedback regulatory mechanism between the family Bacteroidaceae and the genus *Bacteroides*, which may inhibit the onset and progression of GD.

Another mechanism by which the microbiota affects the progression of GD involves the regulation of various metabolites, including metabolites such as short-chain fatty acids and tryptophan (Yan et al., 2024). Our research findings indicate that 56 identified metabolites are significantly enriched in the following metabolic pathways: *De Novo* Triacylglycerol Biosynthesis, Glycerol Phosphate Shuttle, Cardiolipin Biosynthesis, Isoglobo series sphingolipids, and the Biosynthesis of DPAn-6 SPM pathway. Additionally, in-depth analysis revealed that three microbial taxa indirectly influenced GD by affecting five specific metabolic substances. The detailed mechanisms are as follows: *Dialister* demonstrates a positive regulatory effect on GD by down-regulating the plasma mannose to glycerol ratio and up-regulating the levels of 1-(1-enyl-palmitoyl)-glycerophosphocholine (GPC) (p-16:0). Similarly, *Oscillospira* positively influences GD through the modulation of gamma-glutamylthreonine levels. *Dialister* is commonly found in the oral cavity, upper respiratory tract, and intestinal environment of healthy individuals. It has been confirmed to be associated with various human diseases, including bacteremia, periodontitis, rheumatoid arthritis, ankylosing spondylitis and oropharyngeal squamous cell carcinoma (Kaiser et al., 2021; Drago et al., 2013). Although its precise role in the pathological mechanism of GD remains to be elucidated, it is speculated that this strain may contribute to or exacerbate the disease process by releasing lipopolysaccharides, increasing intestinal permeability, and inducing chronic inflammation or immune cell imbalance (Mena-Vázquez et al., 2023). Previous studies have shown a reduction in Dialister and Oscillospira abundance in GD patients (Liu et al., 2024; Yang et al., 2022; Shu et al., 2021), with *Dialister* levels rebounding following anti-thyroid drug treatment (Shu et al., 2021). This contrasts with the findings of the present study. We hypothesize that this discrepancy may stem from variations in sample sources, sample sizes, and dietary habits across different studies.1-(1-enyl-palmitoyl)-GPC (p-16 0), a derivative of glycerophosphatidylcholine with palmitoyl (16 0), is recognized as a potential biomarker for assessing gastric cancer risk. Additionally, this metabolite and its analogous phospholipids can modulate the migration of immune cells and influence the expression of inflammatory factors, thereby contributing to the pathogenesis of autoimmune diseases (Wu et al., 2022; Zheng et al., 2024). Currently, there is no direct evidence linking 1-(1-enyl-palmitoyl)-GPC (p-16:0) to GD. This study explores how Dialister may positively impact GD by regulating plasma levels of 1-(1-enyl-palmitoyl)-GPC (p-16:0). Mannose, a glucose isomer, enhances regulatory T cell (Treg) differentiation in human and murine cells by activating transforming growth factor-β (TGF-β). Given that the proportion of Treg cells in GD patients is lower than that in healthy individuals, it is hypothesized that mannose may ameliorate GD by enhancing the differentiation of Treg cells (Zhang et al., 2017; Chen et al., 2021). Moreover, hyperthyroidism in GD patients can accelerate lipolysis, leading to the conversion of glycerol to triglycerides and subsequent hydrolysis. This study indicates that the ratio of mannose to glycerol may protect the thyroid gland from autoantibodies through this mechanism, thereby preventing or alleviating symptoms of GD. The *Dialister* strain may exert a distinct positive effect on GD by disrupting this protective mechanism, specifically by modulating the ratio of mannose to glycerol. *Oscillospira* exerts a positive effect on GD through the modulation of Gamma-glutamylthreonine levels. Although direct evidence linking *Oscillospira* to hyperthyroidism is lacking, it is noteworthy that patients with GD frequently exhibit metabolic disturbances and show an enriched abundance of *Dialister* compared to healthy individuals. Given the reported positive correlation between *Oscillospira* and *Dialister*, along with the negative correlation between *Oscillospira* and body mass index (BMI), and the significant enrichment of *Dialister* in the feces of GD patients alongside a marked decrease in their BMI, it is plausible to speculate that *Oscillospira* may indirectly associate with hyperthyroidism by influencing the host’s metabolic status (Chen et al., 2020; Konikoff and Gophna, 2016). Furthermore, Gamma-glutamylthreonine, a dipeptide composed of γ-glutamyl and threonine, is a proteolytic breakdown product of larger proteins and may play a role in cellular antioxidant defense mechanisms within the glutathione (GSH) metabolic pathway, thereby protecting cells from oxidative stress damage (Kozlov et al., 2024). In untreated hyperthyroid patients, elevated levels of oxidative stress parameters have been reported in serum, plasma, and erythrocytes compared to normal controls (Alicigüzel et al., 2001). Our study suggests that Gamma-glutamylthreonine may ameliorate GD by participating in the antioxidant defense mechanisms of thyroid cells, while Oscillospira may positively influences GD by potentially attenuating the protective effects of Gamma-glutamylthreonine.

In the current study, we identified four target genes that are regulated by different gut microbiota: *TAGAP*, *HERC3*, *NCEH1*, and *LYN*. T cell activation Rho GTPase activating protein (TAGAP) is a GTPase activating protein linked to susceptibility in various autoimmune diseases, such as Crohn’s disease, psoriasis, and rheumatoid arthritis, due to numerous SNPs located near or within the *TAGAP* gene (Franke et al., 2010; Tsoi et al., 2012; Chatzikyriakidou et al., 2013). TAGAP mRNA expression levels positively correlate with Th17 cell abundance in PBMCs (Chen et al., 2020). Moreover, broad-spectrum tyrosine kinase inhibitors can inhibit Th17 and Th1 cell polarization and thus inhibit the progression of GD. Therefore, it is speculated that *TAGAP* is highly expressed in PBMCs of GD and is involved in the occurrence and development of GD. *HERC3* (HECT and RCC1-like domain-containing protein 3) is a member of the HERC protein family. It has ubiquitin ligase activity and affects the activation of immune cells (such as B cells and T cells) by regulating the IFN signaling pathway or the NF-κB pathway (Zhang et al., 2022). In addition, it is also expressed in intestinal epithelial cells, thyroid and immune cells. The results of this study showed that PBMCs of GD mice highly expressed *HERC3*, indicating that it may be involved in the progression of GD by affecting immune cells. The enzyme encoded by *NCEH1* is involved in cholesterol metabolism, and its main function is to hydrolyze intracellular cholesterol esters and release free cholesterol. Its activity affects: Abnormal cholesterol metabolism may change the function or membrane fluidity of immune cells (such as T cells and B cells) and affect signal transduction. *Lyn* is a member of the intracellular membrane-associated tyrosine kinase (SFK) Src family. The core of Graves’ disease lies in the B cell-mediated autoimmune response: after recognizing thyroid autoantigens, B cells differentiate into plasma cells and secrete pathogenic autoantibodies TRAb. Lyn is one of the first kinases recruited and activated when the B cell receptor recognizes an antigen. It phosphorylates ITAM motifs in the BCR complex, recruits and activates downstream signaling molecules (such as Syk), and initiates a signaling cascade for B cell activation, proliferation, and differentiation (including antibody production) (Xu et al., 2005). Together, these results highlight the functional role of these four genes in the gut-thyroid axis interaction.

The study is subject to several limitations. The study primarily involved individuals of European descent, so caution is advised when applying these findings to non-European populations. Second, we employed a less stringent criterion (1 × 10^^5^) to determine the instrumental variable for partial exposure. Nonetheless, it is reassuring that the F-statistic exceeds 10, indicating that weak instrumental variable bias is likely minimal. Third, our findings are theoretical and lack validation through clinical or animal experiments, leaving the specific mechanisms uncertain. Further cell and animal experiments are necessary to elucidate these mechanisms. We will validate the results’ robustness at the population level using randomized clinical trials. Our findings indicate that plasma metabolite furanol sulfate levels mediated only 19.94% of the effect, suggesting the need for further research to identify additional mediators.

## Conclusion

This study established the causal relationships between the progression of GD and 8 gut microbiota taxa as well as 56 metabolites. Potentially enriched signaling pathways were explored and the target genes under regulation were identified. The causal effect of Dialister on GD risk was mediated by the mannose to glycerol ratio and 1-(1-enyl-palmitoyl)-GPC (p-16:0). In addition, *γ*-glutamylthreonine mediated the causal effect of Oscillospira on GD risk. In addition, the ratios of acetylcarnitine (C2) to propionylcarnitine (C3) and adenosine 5′-diphosphate (ADP) to ornithine mediated the causal effect of MollicutesRF9 on GD. Targeting the expression of *TAGAP*, *HERC3*, *NCEH1*, and *LYN* presents a promising strategy for enhancing GD management. Future research should aim to validate it in different cohorts, as well as use GD animal models and *in vitro* cell experiments to more thoroughly verify and elucidate the pathophysiological roles and the molecular mechanisms related to these gut microbiota, metabolites, and target genes, which will also facilitate the development of new therapeutic interventions for GD.

## Data Availability

The original contributions presented in the study are included in the article/Supplementary material, further inquiries can be directed to the corresponding author.

## Footnotes

1
https://github.com/AnqiWang2021/RBMR

2
https://www.metaboanalyst.ca/
