# Supplementary material for: Integrative analysis of gut microbiota, plasma metabolome, and gene expression identifies causal mediators in Graves’ disease pathogenesis
Source: Front Microbiol. 2026 Aug 3;17:1704766. doi: 10.3389/fmicb.2026.1704766 (PMC13478361; doi:10.3389/fmicb.2026.1704766)
Supplement: Supplementary file 1 [file Supplementary_file_1.docx]

## Supplementary Materials

**Table S1 Primer used for qRT-PCR**

| **Gene name** | Primer | Sequence |
| --- | --- | --- |
| TAGAP | Forward primer | TTCACCACAAAGACGGACAA |
| TAGAP | Reverse primer | GAGAGGGCCCAGGTTCCAGA |
| HERC3 | Forward primer | GATCTGCCGAGAAAGCTATGG |
| HERC3 | Reverse primer | TTTGCCACCACACACCTTTAG |
| NCEH1 | Forward primer | TCAGGGAGAGCAACACCTTC |
| NCEH1 | Reverse primer | TGCCTGTCCTCTGTTGACAT |
| LYN | Forward primer | CAGCAACACGAAGCTTACCG |
| LYN | Reverse primer | TCCCAGGTGCAGGTTTCTTC |

**Table S2 Instrumental variables used in MR analysis of the association between gut microbiota and GD**

| **Exposure** | **SNP** | **Exposure** | | | **Outcome** | | | **F value** |
| --- | --- | --- | --- | --- | --- | --- | --- | --- |
|  |  | **β** | **SE** | **P** | **β** | **SE** | **P** |  |
| family.Bacteroidaceae.917 | rs11585893 | -0.0741 | 0.0148 | 1.80E-06 | 0.0748 | 0.0315 | 0.0175 | 24.3134 |
| family.Bacteroidaceae.917 | rs13207588 | -0.0592 | 0.0131 | 7.49E-06 | 0.0446 | 0.0340 | 0.1892 | 24.3116 |
| family.Bacteroidaceae.917 | rs1340391 | -0.0592 | 0.0132 | 6.73E-06 | 0.0235 | 0.0391 | 0.5471 | 24.3098 |
| family.Bacteroidaceae.917 | rs17619981 | 0.0881 | 0.0187 | 2.69E-06 | -0.0162 | 0.0392 | 0.6797 | 24.3089 |
| family.Bacteroidaceae.id.917 | rs2023437 | -0.0782 | 0.0168 | 5.02E-06 | 0.0305 | 0.0409 | 0.4552 | 24.3030 |
| family.Bacteroidaceae.id.917 | rs2366421 | -0.0528 | 0.0117 | 7.65E-06 | -0.0171 | 0.0348 | 0.6224 | 24.3021 |
| family.Bacteroidaceae.id.917 | rs28757219 | 0.0818 | 0.0170 | 1.29E-06 | -0.0312 | 0.0409 | 0.4452 | 24.3021 |
| family.Bacteroidaceae.id.917 | rs495004 | -0.0607 | 0.0130 | 3.42E-06 | -0.0209 | 0.0319 | 0.5128 | 24.3021 |
| family.Bacteroidaceae.id.917 | rs66710942 | 0.0488 | 0.0107 | 5.86E-06 | -0.0001 | 0.0268 | 0.9980 | 24.2885 |
| family.Bacteroidaceae.id.917 | rs6795673 | 0.0539 | 0.0105 | 3.38E-07 | -0.0224 | 0.0268 | 0.4036 | 24.2846 |
| family.Bacteroidaceae.id.917 | rs9507307 | 0.0604 | 0.0129 | 2.13E-06 | 0.0199 | 0.0313 | 0.5257 | 24.2753 |
| genus.Bacteroides.id.918 | rs11585893 | -0.0741 | 0.0148 | 1.80E-06 | 0.0748 | 0.0315 | 0.0175 | 21.7457 |
| genus.Bacteroides.id.918 | rs13207588 | -0.0592 | 0.0131 | 7.49E-06 | 0.0446 | 0.0340 | 0.1892 | 21.7440 |
| genus.Bacteroides.id.918 | rs1340391 | -0.0592 | 0.0132 | 6.73E-06 | 0.0235 | 0.0391 | 0.5471 | 21.7390 |
| genus.Bacteroides.id.918 | rs17619981 | 0.0881 | 0.0187 | 2.69E-06 | -0.0162 | 0.0392 | 0.6797 | 21.7390 |
| genus.Bacteroides.id.918 | rs2023437 | -0.0782 | 0.0168 | 5.02E-06 | 0.0305 | 0.0409 | 0.4552 | 21.7369 |
| genus.Bacteroides.id.918 | rs2366421 | -0.0528 | 0.0117 | 7.65E-06 | -0.0171 | 0.0348 | 0.6224 | 21.7351 |
| genus.Bacteroides.id.918 | rs28757219 | 0.0818 | 0.0170 | 1.29E-06 | -0.0312 | 0.0409 | 0.4452 | 21.7306 |
| genus.Bacteroides.id.918 | rs495004 | -0.0607 | 0.0130 | 3.42E-06 | -0.0209 | 0.0319 | 0.5128 | 21.7304 |
| genus.Bacteroides.id.918 | rs66710942 | 0.0488 | 0.0107 | 5.86E-06 | -0.0001 | 0.0268 | 0.9980 | 21.7301 |
| genus.Bacteroides.id.918 | rs6795673 | 0.0539 | 0.0105 | 3.38E-07 | -0.0224 | 0.0268 | 0.4036 | 21.7294 |
| genus.Bacteroides.id.918 | rs9507307 | 0.0604 | 0.0129 | 2.13E-06 | 0.0199 | 0.0313 | 0.5257 | 21.7284 |
| genus.Bifidobacterium.id.436 | rs10841473 | -0.0624 | 0.0129 | 1.65E-06 | -0.0290 | 0.0319 | 0.3632 | 21.6955 |
| genus.Bifidobacterium.id.436 | rs12022129 | 0.0619 | 0.0139 | 8.00E-06 | -0.0044 | 0.0301 | 0.8833 | 21.6912 |
| genus.Bifidobacterium.id.436 | rs182549 | 0.1197 | 0.0127 | 1.28E-20 | 0.0375 | 0.0276 | 0.1740 | 21.6857 |
| genus.Bifidobacterium.id.436 | rs2491158 | 0.0713 | 0.0160 | 8.05E-06 | 0.0256 | 0.0394 | 0.5157 | 21.6844 |
| genus.Bifidobacterium.id.436 | rs2686790 | 0.0707 | 0.0158 | 7.50E-06 | -0.0693 | 0.0375 | 0.0649 | 21.6787 |
| genus.Bifidobacterium.id.436 | rs4567981 | 0.0562 | 0.0118 | 1.93E-06 | -0.0408 | 0.0270 | 0.1302 | 21.6750 |
| genus.Bifidobacterium.id.436 | rs540489 | -0.0638 | 0.0139 | 5.19E-06 | -0.0338 | 0.0351 | 0.3347 | 21.6736 |
| genus.Bifidobacterium.id.436 | rs55888705 | 0.0546 | 0.0121 | 6.67E-06 | -0.0362 | 0.0297 | 0.2229 | 21.6719 |
| genus.Bifidobacterium.id.436 | rs56108664 | 0.0730 | 0.0158 | 2.44E-06 | 0.0647 | 0.0398 | 0.1040 | 21.6693 |
| genus.Bifidobacterium.id.436 | rs5746486 | -0.0536 | 0.0121 | 9.00E-06 | -0.0227 | 0.0275 | 0.4091 | 21.6679 |
| genus.Bifidobacterium.id.436 | rs62181700 | -0.0625 | 0.0131 | 2.17E-06 | -0.0299 | 0.0311 | 0.3359 | 21.6661 |
| genus.Bifidobacterium.id.436 | rs7322849 | 0.1124 | 0.0202 | 1.08E-08 | 0.1084 | 0.0469 | 0.0207 | 21.6649 |
| genus.Bifidobacterium.id.436 | rs75344046 | 0.2324 | 0.0506 | 4.86E-06 | 0.0527 | 0.0634 | 0.4063 | 21.6634 |
| genus.Bifidobacterium.id.436 | rs76671854 | -0.0846 | 0.0184 | 3.96E-06 | -0.0509 | 0.0406 | 0.2094 | 21.6627 |
| genus.Bifidobacterium.id.436 | rs857444 | 0.0558 | 0.0121 | 3.57E-06 | 0.0648 | 0.0278 | 0.0197 | 21.6583 |
| genus.Catenibacterium.id.2153 | rs12404911 | 0.1407 | 0.0304 | 2.80E-06 | 0.0427 | 0.0343 | 0.2132 | 21.4606 |
| genus.Catenibacterium.id.2153 | rs212393 | -0.1353 | 0.0286 | 3.62E-06 | -0.0948 | 0.0335 | 0.0047 | 21.4554 |
| genus.Catenibacterium.id.2153 | rs73128290 | 0.1297 | 0.0285 | 4.29E-06 | 0.0377 | 0.0293 | 0.1977 | 21.4554 |
| genus.Catenibacterium.id.2153 | rs7742829 | 0.1141 | 0.0251 | 5.61E-06 | 0.0098 | 0.0270 | 0.7172 | 21.4551 |
| genus.Dialister.id.2183 | rs10138457 | -0.1131 | 0.0262 | 7.88E-06 | 0.0093 | 0.0464 | 0.8410 | 21.2454 |
| genus.Dialister.id.2183 | rs10938938 | -0.0774 | 0.0171 | 7.37E-06 | -0.0052 | 0.0366 | 0.8869 | 21.2454 |
| genus.Dialister.id.2183 | rs11071887 | 0.0662 | 0.0146 | 5.91E-06 | 0.0370 | 0.0287 | 0.1976 | 21.2454 |
| genus.Dialister.id.2183 | rs11166701 | -0.0655 | 0.0132 | 5.51E-07 | -0.0280 | 0.0269 | 0.2972 | 21.2446 |
| genus.Dialister.id.2183 | rs2314294 | 0.0866 | 0.0194 | 8.08E-06 | 0.0471 | 0.0392 | 0.2291 | 21.2435 |
| genus.Dialister.id.2183 | rs2435610 | 0.0647 | 0.0143 | 5.93E-06 | 0.0188 | 0.0308 | 0.5428 | 21.2381 |
| genus.Dialister.id.2183 | rs4747450 | 0.0669 | 0.0148 | 5.84E-06 | 0.0257 | 0.0323 | 0.4263 | 21.2377 |
| genus.Dialister.id.2183 | rs4753063 | -0.0596 | 0.0130 | 4.86E-06 | -0.0390 | 0.0269 | 0.1473 | 21.2361 |
| genus.Dialister.id.2183 | rs517089 | 0.0762 | 0.0170 | 5.14E-06 | -0.0259 | 0.0356 | 0.4662 | 21.2333 |
| genus.Dialister.id.2183 | rs75416973 | 0.0727 | 0.0165 | 9.46E-06 | -0.0773 | 0.0325 | 0.0173 | 21.2333 |
| genus.Dialister.id.2183 | rs764177 | -0.0601 | 0.0135 | 9.61E-06 | -0.0107 | 0.0279 | 0.7014 | 21.2330 |
| genus.Dialister.id.2183 | rs76680460 | -0.1613 | 0.0364 | 8.19E-06 | -0.1469 | 0.0663 | 0.0267 | 21.2328 |
| genus.Oscillospira.id.2064 | rs12206468 | -0.1330 | 0.0270 | 1.04E-06 | -0.0474 | 0.0503 | 0.3459 | 20.4535 |
| genus.Oscillospira.id.2064 | rs12925026 | 0.1356 | 0.0307 | 9.31E-06 | -0.0445 | 0.0584 | 0.4458 | 20.4522 |
| genus.Oscillospira.id.2064 | rs1954532 | -0.0826 | 0.0175 | 2.27E-06 | -0.0138 | 0.0336 | 0.6808 | 20.4476 |
| genus.Oscillospira.id.2064 | rs28889936 | 0.1141 | 0.0253 | 3.37E-06 | 0.0169 | 0.0455 | 0.7109 | 20.4462 |
| genus.Oscillospira.id.2064 | rs62422654 | 0.0898 | 0.0198 | 6.47E-06 | 0.0486 | 0.0330 | 0.1408 | 20.4452 |
| genus.Oscillospira.id.2064 | rs72866977 | -0.1306 | 0.0282 | 5.63E-06 | -0.0587 | 0.0502 | 0.2424 | 20.4442 |
| genus.Oscillospira.id.2064 | rs73038677 | -0.0832 | 0.0169 | 1.09E-06 | -0.0272 | 0.0389 | 0.4842 | 20.4439 |
| genus.Oscillospira.id.2064 | rs751183 | -0.0774 | 0.0172 | 6.85E-06 | -0.0261 | 0.0346 | 0.4515 | 20.4433 |
| genus.Oscillospira.id.2064 | rs8076323 | 0.0715 | 0.0157 | 5.61E-06 | 0.0414 | 0.0285 | 0.1467 | 20.4421 |
| genus.Ruminiclostridium5.id.11355 | rs10827477 | -0.0547 | 0.0115 | 2.19E-06 | 0.0097 | 0.0281 | 0.7309 | 20.2578 |
| genus.Ruminiclostridium5.id.11355 | rs113753996 | 0.0821 | 0.0174 | 3.99E-06 | 0.0475 | 0.0348 | 0.1722 | 20.2558 |
| genus.Ruminiclostridium5.id.11355 | rs1223978 | 0.0484 | 0.0108 | 8.16E-06 | -0.0310 | 0.0270 | 0.2508 | 20.2539 |
| genus.Ruminiclostridium5.id.11355 | rs1492620 | -0.0831 | 0.0180 | 3.53E-06 | -0.0276 | 0.0406 | 0.4958 | 20.2533 |
| genus.Ruminiclostridium5.id.11355 | rs2286384 | -0.0519 | 0.0107 | 1.44E-06 | -0.0054 | 0.0267 | 0.8387 | 20.2511 |
| genus.Ruminiclostridium5.id.11355 | rs243585 | -0.0586 | 0.0121 | 1.33E-06 | 0.0265 | 0.0301 | 0.3785 | 20.2508 |
| genus.Ruminiclostridium5.id.11355 | rs2482038 | 0.0519 | 0.0109 | 1.70E-06 | 0.0162 | 0.0273 | 0.5542 | 20.2479 |
| genus.Ruminiclostridium5.id.11355 | rs2791343 | 0.0517 | 0.0113 | 5.54E-06 | 0.0164 | 0.0273 | 0.5482 | 20.2470 |
| genus.Ruminiclostridium5.id.11355 | rs2801960 | 0.0521 | 0.0115 | 6.21E-06 | 0.0010 | 0.0293 | 0.9737 | 20.2465 |
| genus.Ruminiclostridium5.id.11355 | rs2833828 | 0.0490 | 0.0109 | 6.82E-06 | 0.0420 | 0.0272 | 0.1228 | 20.2463 |
| genus.Ruminiclostridium5.id.11355 | rs4955951 | -0.0714 | 0.0166 | 9.96E-06 | 0.0411 | 0.0414 | 0.3213 | 20.2445 |
| genus.Ruminiclostridium5.id.11355 | rs6121460 | 0.0933 | 0.0199 | 2.64E-06 | 0.0535 | 0.0495 | 0.2799 | 20.2439 |
| genus.Ruminiclostridium5.id.11355 | rs73002572 | 0.1815 | 0.0414 | 8.82E-06 | 0.0353 | 0.0424 | 0.4045 | 20.2439 |
| genus.Ruminiclostridium5.id.11355 | rs79968837 | -0.0950 | 0.0194 | 1.15E-06 | -0.1173 | 0.0601 | 0.0510 | 20.2401 |
| genus.Ruminiclostridium5.id.11355 | rs8053158 | -0.0741 | 0.0159 | 5.90E-06 | -0.0520 | 0.0411 | 0.2057 | 20.2400 |
| order.MollicutesRF9.id.11579 | rs10071529 | 0.1251 | 0.0279 | 8.64E-06 | 0.0067 | 0.0432 | 0.8770 | 19.5075 |
| order.MollicutesRF9.id.11579 | rs11779863 | -0.0773 | 0.0172 | 6.69E-06 | 0.0064 | 0.0370 | 0.8631 | 19.5042 |
| order.MollicutesRF9.id.11579 | rs12566890 | -0.1031 | 0.0242 | 8.11E-06 | -0.0425 | 0.0400 | 0.2873 | 19.5036 |
| order.MollicutesRF9.id.11579 | rs13100746 | 0.0639 | 0.0143 | 7.29E-06 | -0.0017 | 0.0269 | 0.9497 | 19.5015 |
| order.MollicutesRF9.id.11579 | rs17235252 | -0.1220 | 0.0255 | 2.16E-06 | -0.0631 | 0.0415 | 0.1281 | 19.4993 |
| order.MollicutesRF9.id.11579 | rs3932485 | 0.0626 | 0.0142 | 9.93E-06 | 0.0251 | 0.0274 | 0.3608 | 19.4978 |
| order.MollicutesRF9.id.11579 | rs515984 | -0.0875 | 0.0191 | 6.61E-06 | -0.0145 | 0.0417 | 0.7272 | 19.4953 |
| order.MollicutesRF9.id.11579 | rs62188991 | -0.1109 | 0.0241 | 5.27E-06 | -0.0060 | 0.0579 | 0.9172 | 19.4912 |
| order.MollicutesRF9.id.11579 | rs638542 | -0.0706 | 0.0157 | 5.17E-06 | 0.0114 | 0.0295 | 0.6989 | 19.4912 |
| order.MollicutesRF9.id.11579 | rs739151 | 0.0653 | 0.0140 | 3.09E-06 | 0.0157 | 0.0268 | 0.5579 | 19.4912 |
| order.MollicutesRF9.id.11579 | rs74603314 | 0.2308 | 0.0490 | 2.28E-06 | 0.0829 | 0.0683 | 0.2249 | 19.4899 |
| order.MollicutesRF9.id.11579 | rs7706512 | 0.0657 | 0.0139 | 2.27E-06 | -0.0164 | 0.0268 | 0.5419 | 19.4862 |
| order.MollicutesRF9.id.11579 | rs7801843 | -0.0869 | 0.0195 | 9.47E-06 | -0.0303 | 0.0374 | 0.4178 | 19.4724 |
| order.MollicutesRF9.id.11579 | rs7853673 | -0.0624 | 0.0140 | 6.73E-06 | -0.0118 | 0.0268 | 0.6590 | 19.4678 |
| order.MollicutesRF9.id.11579 | rs949341 | 0.0657 | 0.0147 | 7.73E-06 | 0.0500 | 0.0300 | 0.0950 | 19.4678 |

Table S3 The potential causal effects of gut microbiota to GD was calculated by BWMR method.

| **Exposure** | **Outcome** | **method** | **beta** | **Or(95%CI)** | **pval** |
| --- | --- | --- | --- | --- | --- |
| family.Bacteroidaceae.id.917 | GD | BWMR | -0.284 | 0.753(0.544-1.041) | 0.086 |
| genus.Bacteroides.id.918 | GD | BWMR | -0.284 | 0.753(0.544-1.041) | 0.086 |
| genus.Bifidobacterium.id.436 | GD | BWMR | 0.320 | 1.378(1.034-1.835) | **0.029** |
| genus.Catenibacterium.id.2153 | GD | BWMR | 0.355 | 1.426(1.092-1.862) | **0.009** |
| genus.Dialister.id.2183 | GD | BWMR | 0.234 | 1.264(0.931-1.715) | **0.034** |
| genus.Oscillospira.id.2064 | GD | BWMR | 0.312 | 1.366(1.034-1.805) | **0.028** |
| genus.Ruminiclostridium5.id.11355 | GD | BWMR | 0.232 | 1.261(0.972-1.635) | 0.080 |
| order.MollicutesRF9.id.11579 | GD | BWMR | 0.219 | 1.245(1.003-1.546) | **0.047** |

**Table S4 The heterogeneity of gut microbiota instrumental variables**

| **Exposure** | **Outcome** | **Method** | **Q** | **Q_df** | **Q_pval** |
| --- | --- | --- | --- | --- | --- |
| family.Bacteroidaceae.id.917 | GD | MR Egger | 4.8024 | 6 | 0.5694 |
| family.Bacteroidaceae.id.917 | GD | Inverse variance weighted | 5.1649 | 7 | 0.6398 |
| genus.Bacteroides.id.918 | GD | MR Egger | 4.8024 | 6 | 0.5694 |
| genus.Bacteroides.id.918 | GD | Inverse variance weighted | 5.1649 | 7 | 0.6398 |
| genus.Bifidobacterium.id.436 | GD | MR Egger | 16.6107 | 10 | 0.0834 |
| genus.Bifidobacterium.id.436 | GD | Inverse variance weighted | 16.6464 | 11 | 0.1188 |
| genus.Catenibacterium.id.2153 | GD | MR Egger | 1.9127 | 2 | 0.3843 |
| genus.Catenibacterium.id.2153 | GD | Inverse variance weighted | 3.3469 | 3 | 0.3412 |
| genus.Dialister.id.2183 | GD | MR Egger | 13.8082 | 9 | 0.1293 |
| genus.Dialister.id.2183 | GD | Inverse variance weighted | 14.0408 | 10 | 0.1711 |
| genus.Oscillospira.id.2064 | GD | MR Egger | 2.7831 | 6 | 0.8355 |
| genus.Oscillospira.id.2064 | GD | Inverse variance weighted | 3.4791 | 7 | 0.8374 |
| genus.Ruminiclostridium5.id.11355 | GD | MR Egger | 8.6062 | 9 | 0.4744 |
| genus.Ruminiclostridium5.id.11355 | GD | Inverse variance weighted | 10.3882 | 10 | 0.4071 |
| order.MollicutesRF9.id.11579 | GD | MR Egger | 4.8727 | 10 | 0.8995 |
| order.MollicutesRF9.id.11579 | GD | Inverse variance weighted | 5.7826 | 11 | 0.8875 |

**Table S5 Directional horizontal pleiotropy assessed by intercept term in MR Egger regression of the association between gut microbiota and GD**

| **Exposure** | **Outcome** | **Egger intercept** | **SE** | **Pvalue** |
| --- | --- | --- | --- | --- |
| family.Bacteroidaceae.id.917 | GD | 0.0366 | 0.0608 | 0.5691 |
| genus.Bacteroides.id.918 | GD | 0.0366 | 0.0608 | 0.5691 |
| genus.Bifidobacterium.id.436 | GD | -0.0046 | 0.0310 | 0.8863 |
| genus.Catenibacterium.id.2153 | GD | -0.2287 | 0.1909 | 0.3538 |
| genus.Dialister.id.2183 | GD | -0.0194 | 0.0499 | 0.7061 |
| genus.Oscillospira.id.2064 | GD | 0.0478 | 0.0573 | 0.4361 |
| genus.Ruminiclostridium5.id.11355 | GD | -0.0559 | 0.0419 | 0.2147 |
| order.MollicutesRF9.id.11579 | GD | -0.0274 | 0.0287 | 0.3626 |

**Table S6 Heterogeneity analysis of MR results of GD and gut microbiota**

| **Exposure** | **Outcome** | **Method** | **Q** | **Q_df** | **Q_pval** |
| --- | --- | --- | --- | --- | --- |
| GD | class.Actinobacteria | MR Egger | 33.988 | 27 | 0.166 |
|  |  | IVW | 34.069 | 28 | 0.199 |
| GD | family.Bacteroidaceae | MR Egger | 20.315 | 27 | 0.817 |
|  |  | IVW | 20.787 | 28 | 0.834 |
| GD | family.Bifidobacteriaceae | MR Egger | 20.315 | 27 | 0.817 |
|  |  | IVW | 20.787 | 28 | 0.834 |
| GD | family.Peptococcaceae | MR Egger | 23.874 | 25 | 0.527 |
|  |  | IVW | 24.118 | 26 | 0.569 |
| GD | family.Streptococcaceae | MR Egger | 28.015 | 26 | 0.358 |
|  |  | IVW | 29.602 | 27 | 0.332 |
| GD | genus.Eubacterium hallii group | MR Egger | 37.785 | 27 | 0.081 |
|  |  | IVW | 44.741 | 28 | 0.023 |
| GD | genus.Ruminococcustorquesgroup | MR Egger | 25.422 | 27 | 0.551 |
|  |  | IVW | 25.586 | 28 | 0.596 |
| GD | genus.Adlercreutzia | MR Egger | 26.445 | 25 | 0.384 |
|  |  | IVW | 26.456 | 26 | 0.438 |
| GD | genus.Bacteroides | MR Egger | 26.739 | 27 | 0.478 |
|  |  | IVW | 27.208 | 28 | 0.507 |
| GD | genus.Bifidobacterium | MR Egger | 19.276 | 27 | 0.860 |
|  |  | IVW | 19.552 | 28 | 0.880 |
| GD | genus.Lachnospiraceae NK4A136 | MR Egger | 28.645 | 26 | 0.327 |
|  |  | IVW | 32.769 | 27 | 0.205 |
| GD | genus.Lachnospiraceae UCG008 | MR Egger | 19.542 | 25 | 0.770 |
|  |  | IVW | 21.024 | 26 | 0.741 |
| GD | genus.Roseburia | MR Egger | 30.940 | 27 | 0.274 |
|  |  | IVW | 31.046 | 28 | 0.315 |
| GD | genus.Ruminococcaceae UCG013 | MR Egger | 23.873 | 26 | 0.583 |
|  |  | IVW | 25.415 | 27 | 0.551 |
| GD | genus.Streptococcus | MR Egger | 28.703 | 26 | 0.325 |
|  |  | IVW | 30.459 | 27 | 0.294 |
| GD | genus.Veillonella | MR Egger | 22.078 | 25 | 0.631 |
|  |  | IVW | 30.969 | 26 | 0.229 |
| GD | order.Bifidobacteriales | MR Egger | 20.315 | 27 | 0.817 |
|  |  | IVW | 20.787 | 28 | 0.834 |
| GD | phylum.Actinobacteria | MR Egger | 26.503 | 27 | 0.491 |
|  |  | IVW | 27.535 | 28 | 0.489 |

**Table S7 Directional horizontal pleiotropy assessed by intercept term in MR Egger regression of the association between GD and gut microbiota**

| **Exposure** | **Outcome** | **Egger intercept** | **SE** | **Pvalue** |
| --- | --- | --- | --- | --- |
| GD | class.Actinobacteria | 0.003 | 0.010 | 0.801 |
| GD | family.Bacteroidaceae | 0.006 | 0.008 | 0.499 |
| GD | family.Bifidobacteriaceae | 0.006 | 0.009 | 0.498 |
| GD | family.Peptococcaceae | -0.006 | 0.011 | 0.626 |
| GD | family.Streptococcaceae | 0.011 | 0.009 | 0.236 |
| GD | genus.Eubacterium hallii group | 0.024 | 0.011 | 0.034 |
| GD | genus.Ruminococcus torques group | 0.003 | 0.009 | 0.689 |
| GD | genus.Adlercreutzia | -0.001 | 0.013 | 0.919 |
| GD | genus.Bacteroides | 0.006 | 0.008 | 0.499 |
| GD | genus.Bifidobacterium | 0.005 | 0.009 | 0.604 |
| GD | genus.Lachnospiraceae NK4A136 | -0.018 | 0.009 | 0.064 |
| GD | genus.Lachnospiraceae UCG008 | 0.017 | 0.014 | 0.235 |
| GD | genus.Roseburia | 0.003 | 0.009 | 0.764 |
| GD | genus.Ruminococcaceae UCG013 | 0.011 | 0.009 | 0.225 |
| GD | genus.Streptococcus | 0.012 | 0.010 | 0.218 |
| GD | genus.Veillonella | -0.036 | 0.012 | 0.006 |
| GD | order.Bifidobacteriales | 0.006 | 0.009 | 0.498 |
| GD | phylum.Actinobacteria | 0.009 | 0.009 | 0.319 |

**Table S8 IVW of metabolites and GD**

| Exposure | Outcome | Method | nSNP | Pvalue | OR |
| --- | --- | --- | --- | --- | --- |
|  |  |  |  |  |  |
| Glycerol 3-phosphate levels | GD | IVW | 29 | 0.0378 | 1.1555 |
| 3-indoxyl sulfate levels | GD | IVW | 21 | 0.0201 | 1.2380 |
| Docosapentaenoate (n6 DPA; 22:5n6) levels | GD | IVW | 14 | 0.0248 | 0.8144 |
| 1-ribosyl-imidazoleacetate levels | GD | IVW | 37 | 0.0460 | 1.1229 |
| Mannitol/sorbitol levels | GD | IVW | 24 | 0.0041 | 1.2545 |
| Hexanoylglutamine levels | GD | IVW | 22 | 0.0000 | 1.2912 |
| 4-allylphenol sulfate levels | GD | IVW | 22 | 0.0009 | 1.2736 |
| 21-hydroxypregnenolone disulfate levels | GD | IVW | 36 | 0.0213 | 0.8853 |
| 2,3-dihydroxyisovalerate levels | GD | IVW | 23 | 0.0063 | 1.1998 |
| Isoursodeoxycholate levels | GD | IVW | 19 | 0.0145 | 1.2058 |
| 2-stearoyl-GPE (18:0) levels | GD | IVW | 30 | 0.0298 | 1.1407 |
| Dimethyl sulfone levels | GD | IVW | 23 | 0.0006 | 0.7763 |
| S-allylcysteine levels | GD | IVW | 17 | 0.0032 | 1.2469 |
| 4-methylguaiacol sulfate levels | GD | IVW | 11 | 0.0155 | 0.7901 |
| 1-palmitoyl-2-stearoyl-gpc (16:0/18:0) levels | GD | IVW | 29 | 0.0187 | 1.1739 |
| 1-(1-enyl-palmitoyl)-2-oleoyl-GPE (p-16:0/18:1) levels | GD | IVW | 29 | 0.0389 | 0.8699 |
| Pimeloylcarnitine/3-methyladipoylcarnitine (C7-DC) levels | GD | IVW | 26 | 0.0489 | 0.8565 |
| N-stearoyl-sphingadienine (d18:2/18:0) levels | GD | IVW | 20 | 0.0098 | 0.8315 |
| Ethyl alpha-glucopyranoside levels | GD | IVW | 29 | 0.0448 | 0.8837 |
| Glycosyl ceramide (d18:1/20:0, d16:1/22:0) levels | GD | IVW | 31 | 0.0343 | 0.8969 |
| Cortolone glucuronide (1) levels | GD | IVW | 23 | 0.0217 | 0.8531 |
| 5-hydroxy-2-methylpyridine sulfate levels | GD | IVW | 18 | 0.0387 | 1.1765 |
| 4-acetylcatechol sulfate (1) levels | GD | IVW | 18 | 0.0291 | 0.9176 |
| Pregnenetriol sulfate levels | GD | IVW | 33 | 0.0123 | 0.8850 |
| 3-hydroxyoctanoylcarnitine (1) levels | GD | IVW | 17 | 0.0489 | 1.2058 |
| Methyl indole-3-acetate levels | GD | IVW | 20 | 0.0118 | 1.2092 |
| Gamma-glutamylglutamine levels | GD | IVW | 29 | 0.0062 | 0.8571 |
| 2-hydroxystearate levels | GD | IVW | 22 | 0.0063 | 1.2398 |
| 1-palmitoyl-2-linoleoyl-gpc (16:0/18:2) levels | GD | IVW | 35 | 0.0446 | 1.1111 |
| Stearoyl sphingomyelin (d18:1/18:0) levels | GD | IVW | 32 | 0.0054 | 0.8466 |
| X-11315 levels | GD | IVW | 24 | 0.0350 | 1.1291 |
| X-12104 levels | GD | IVW | 13 | 0.0445 | 0.8247 |
| X-12013 levels | GD | IVW | 22 | 0.0225 | 1.1671 |
| X-12812 levels | GD | IVW | 18 | 0.0100 | 1.2322 |
| X-12839 levels | GD | IVW | 28 | 0.0472 | 1.1294 |
| X-15486 levels | GD | IVW | 15 | 0.0465 | 1.1842 |
| X-17328 levels | GD | IVW | 23 | 0.0391 | 0.8727 |
| X-17010 levels | GD | IVW | 19 | 0.0157 | 0.8320 |
| X-17676 levels | GD | IVW | 30 | 0.0488 | 0.8792 |
| X-17690 levels | GD | IVW | 27 | 0.0049 | 1.2215 |
| X-21441 levels | GD | IVW | 33 | 0.0491 | 1.0635 |
| X-23654 levels | GD | IVW | 38 | 0.0331 | 0.8839 |
| X-23997 levels | GD | IVW | 20 | 0.0397 | 1.1969 |
| X-24531 levels | GD | IVW | 22 | 0.0046 | 0.8212 |
| Adenosine 5'-diphosphate (ADP) to fructose ratio | GD | IVW | 30 | 0.0333 | 1.1039 |
| Adenosine 5'-diphosphate (ADP) to 2'-deoxyuridine ratio | GD | IVW | 21 | 0.0264 | 1.1415 |
| Phosphate to alanine ratio | GD | IVW | 18 | 0.0157 | 1.2411 |
| Spermidine to phosphate ratio | GD | IVW | 19 | 0.0125 | 0.8282 |
| Adenosine 5'-diphosphate (ADP) to oxalate (ethanedioate) ratio | GD | IVW | 21 | 0.0222 | 1.0864 |
| Cysteinylglycine to glutamate ratio | GD | IVW | 25 | 0.0488 | 0.8773 |
| Adenosine 5'-monophosphate (AMP) to glutamate ratio | GD | IVW | 22 | 0.0244 | 1.2129 |
| Citrate to 4-hydroxyphenylpyruvate ratio | GD | IVW | 19 | 0.0454 | 0.8541 |
| Inosine to EDTA ratio | GD | IVW | 24 | 0.0298 | 0.8830 |
| Mannose to glycerol ratio | GD | IVW | 19 | 0.0322 | 0.8680 |
| Mannose to mannitol to sorbitol ratio | GD | IVW | 21 | 0.0022 | 0.7754 |
| Acetylcarnitine (C2) to propionylcarnitine (C3) ratio | GD | IVW | 19 | 0.0395 | 0.8486 |
| Proline to trans-4-hydroxyproline ratio | GD | IVW | 24 | 0.0099 | 1.2158 |
| Adenosine 5'-diphosphate (ADP) to ornithine ratio | GD | IVW | 23 | 0.0386 | 0.8970 |
| Fructose to maltose ratio | GD | IVW | 22 | 0.0307 | 0.8588 |

**Table S9 Heterogeneity analyse of MR result between metabolites and GD**

| **Exposure** | **Outcome** | **Method** | **Q** | **Q_df** | **Q_pval** |
| --- | --- | --- | --- | --- | --- |
| Glycerol 3-phosphate levels | GD | MR Egger | 38.7082 | 27 | 0.0673 |
| Glycerol 3-phosphate levels | GD | Inverse variance weighted | 38.8121 | 28 | 0.0840 |
| 3-indoxyl sulfate levels | GD | MR Egger | 21.5595 | 19 | 0.3067 |
| 3-indoxyl sulfate levels | GD | Inverse variance weighted | 26.7812 | 20 | 0.1415 |
| Docosapentaenoate (n6 DPA; 22:5n6) levels | GD | MR Egger | 7.4282 | 12 | 0.8281 |
| Docosapentaenoate (n6 DPA; 22:5n6) levels | GD | Inverse variance weighted | 7.4586 | 13 | 0.8770 |
| 1-ribosyl-imidazoleacetate levels | GD | MR Egger | 24.6736 | 35 | 0.9032 |
| 1-ribosyl-imidazoleacetate levels | GD | Inverse variance weighted | 25.5331 | 36 | 0.9028 |
| Mannitol/sorbitol levels | GD | MR Egger | 21.1855 | 22 | 0.5093 |
| Mannitol/sorbitol levels | GD | Inverse variance weighted | 21.9594 | 23 | 0.5227 |
| Hexanoylglutamine levels | GD | MR Egger | 20.8867 | 20 | 0.4038 |
| Hexanoylglutamine levels | GD | Inverse variance weighted | 21.0082 | 21 | 0.4584 |
| 4-allylphenol sulfate levels | GD | MR Egger | 19.7488 | 20 | 0.4737 |
| 4-allylphenol sulfate levels | GD | Inverse variance weighted | 23.7243 | 21 | 0.3066 |
| 21-hydroxypregnenolone disulfate levels | GD | MR Egger | 35.2001 | 34 | 0.4112 |
| 21-hydroxypregnenolone disulfate levels | GD | Inverse variance weighted | 35.2556 | 35 | 0.4561 |
| 2,3-dihydroxyisovalerate levels | GD | MR Egger | 17.2114 | 21 | 0.6982 |
| 2,3-dihydroxyisovalerate levels | GD | Inverse variance weighted | 17.4010 | 22 | 0.7409 |
| Isoursodeoxycholate levels | GD | MR Egger | 9.4977 | 17 | 0.9233 |
| Isoursodeoxycholate levels | GD | Inverse variance weighted | 9.7242 | 18 | 0.9405 |
| 2-stearoyl-GPE (18:0) levels | GD | MR Egger | 29.6737 | 28 | 0.3790 |
| 2-stearoyl-GPE (18:0) levels | GD | Inverse variance weighted | 30.0163 | 29 | 0.4132 |
| Dimethyl sulfone levels | GD | MR Egger | 23.7881 | 21 | 0.3034 |
| Dimethyl sulfone levels | GD | Inverse variance weighted | 24.0380 | 22 | 0.3452 |
| S-allylcysteine levels | GD | MR Egger | 9.5464 | 15 | 0.8473 |
| S-allylcysteine levels | GD | Inverse variance weighted | 9.7598 | 16 | 0.8789 |
| 4-methylguaiacol sulfate levels | GD | MR Egger | 7.1934 | 9 | 0.6170 |
| 4-methylguaiacol sulfate levels | GD | Inverse variance weighted | 7.3441 | 10 | 0.6926 |
| 1-palmitoyl-2-stearoyl-gpc (16:0/18:0) levels | GD | MR Egger | 31.9259 | 27 | 0.2348 |
| 1-palmitoyl-2-stearoyl-gpc (16:0/18:0) levels | GD | Inverse variance weighted | 36.4939 | 28 | 0.1304 |
| 1-(1-enyl-palmitoyl)-2-oleoyl-GPE (p-16:0/18:1) levels | GD | MR Egger | 30.8120 | 27 | 0.2789 |
| 1-(1-enyl-palmitoyl)-2-oleoyl-GPE (p-16:0/18:1) levels | GD | Inverse variance weighted | 31.8828 | 28 | 0.2793 |
| Pimeloylcarnitine/3-methyladipoylcarnitine (C7-DC) levels | GD | MR Egger | 41.2389 | 24 | 0.0157 |
| Pimeloylcarnitine/3-methyladipoylcarnitine (C7-DC) levels | GD | Inverse variance weighted | 42.3348 | 25 | 0.0165 |
| N-stearoyl-sphingadienine (d18:2/18:0) levels | GD | MR Egger | 11.4952 | 18 | 0.8722 |
| N-stearoyl-sphingadienine (d18:2/18:0) levels | GD | Inverse variance weighted | 14.0672 | 19 | 0.7798 |
| Ethyl alpha-glucopyranoside levels | GD | MR Egger | 16.6131 | 27 | 0.9402 |
| Ethyl alpha-glucopyranoside levels | GD | Inverse variance weighted | 22.9218 | 28 | 0.7369 |
| Glycosyl ceramide (d18:1/20:0, d16:1/22:0) levels | GD | MR Egger | 23.9338 | 29 | 0.7322 |
| Glycosyl ceramide (d18:1/20:0, d16:1/22:0) levels | GD | Inverse variance weighted | 23.9881 | 30 | 0.7726 |
| Cortolone glucuronide (1) levels | GD | MR Egger | 16.3311 | 21 | 0.7508 |
| Cortolone glucuronide (1) levels | GD | Inverse variance weighted | 17.1320 | 22 | 0.7560 |
| 5-hydroxy-2-methylpyridine sulfate levels | GD | MR Egger | 14.8459 | 16 | 0.5360 |
| 5-hydroxy-2-methylpyridine sulfate levels | GD | Inverse variance weighted | 15.1409 | 17 | 0.5853 |
| 4-acetylcatechol sulfate (1) levels | GD | MR Egger | 12.2736 | 16 | 0.7249 |
| 4-acetylcatechol sulfate (1) levels | GD | Inverse variance weighted | 12.3067 | 17 | 0.7812 |
| Pregnenetriol sulfate levels | GD | MR Egger | 30.0018 | 31 | 0.5172 |
| Pregnenetriol sulfate levels | GD | Inverse variance weighted | 30.0202 | 32 | 0.5671 |
| 3-hydroxyoctanoylcarnitine (1) levels | GD | MR Egger | 6.3764 | 15 | 0.9727 |
| 3-hydroxyoctanoylcarnitine (1) levels | GD | Inverse variance weighted | 9.4810 | 16 | 0.8923 |
| Methyl indole-3-acetate levels | GD | MR Egger | 17.6173 | 18 | 0.4811 |
| Methyl indole-3-acetate levels | GD | Inverse variance weighted | 21.4893 | 19 | 0.3104 |
| Gamma-glutamylglutamine levels | GD | MR Egger | 27.1656 | 27 | 0.4549 |
| Gamma-glutamylglutamine levels | GD | Inverse variance weighted | 27.4793 | 28 | 0.4923 |
| 2-hydroxystearate levels | GD | MR Egger | 17.0399 | 20 | 0.6504 |
| 2-hydroxystearate levels | GD | Inverse variance weighted | 17.1566 | 21 | 0.7016 |
| 1-palmitoyl-2-linoleoyl-gpc (16:0/18:2) levels | GD | MR Egger | 36.2606 | 33 | 0.3190 |
| 1-palmitoyl-2-linoleoyl-gpc (16:0/18:2) levels | GD | Inverse variance weighted | 39.5501 | 34 | 0.2359 |
| Stearoyl sphingomyelin (d18:1/18:0) levels | GD | MR Egger | 34.7333 | 30 | 0.2525 |
| Stearoyl sphingomyelin (d18:1/18:0) levels | GD | Inverse variance weighted | 36.4869 | 31 | 0.2287 |
| Adenosine 5'-diphosphate (ADP) to fructose ratio | GD | MR Egger | 28.8509 | 28 | 0.4201 |
| Adenosine 5'-diphosphate (ADP) to fructose ratio | GD | Inverse variance weighted | 30.7126 | 29 | 0.3791 |
| Adenosine 5'-diphosphate (ADP) to 2'-deoxyuridine ratio | GD | MR Egger | 16.3128 | 19 | 0.6363 |
| Adenosine 5'-diphosphate (ADP) to 2'-deoxyuridine ratio | GD | Inverse variance weighted | 17.6799 | 20 | 0.6085 |
| Phosphate to alanine ratio | GD | MR Egger | 10.6510 | 16 | 0.8305 |
| Phosphate to alanine ratio | GD | Inverse variance weighted | 13.8947 | 17 | 0.6745 |
| Spermidine to phosphate ratio | GD | MR Egger | 11.2515 | 17 | 0.8432 |
| Spermidine to phosphate ratio | GD | Inverse variance weighted | 13.1850 | 18 | 0.7805 |
| Adenosine 5'-diphosphate (ADP) to oxalate (ethanedioate) ratio | GD | MR Egger | 17.2647 | 19 | 0.5719 |
| Adenosine 5'-diphosphate (ADP) to oxalate (ethanedioate) ratio | GD | Inverse variance weighted | 18.0325 | 20 | 0.5853 |
| Cysteinylglycine to glutamate ratio | GD | MR Egger | 21.1790 | 23 | 0.5702 |
| Cysteinylglycine to glutamate ratio | GD | Inverse variance weighted | 21.1824 | 24 | 0.6279 |
| Adenosine 5'-monophosphate (AMP) to glutamate ratio | GD | MR Egger | 16.6204 | 20 | 0.6775 |
| Adenosine 5'-monophosphate (AMP) to glutamate ratio | GD | Inverse variance weighted | 17.4091 | 21 | 0.6860 |
| Citrate to 4-hydroxyphenylpyruvate ratio | GD | MR Egger | 7.5562 | 17 | 0.9751 |
| Citrate to 4-hydroxyphenylpyruvate ratio | GD | Inverse variance weighted | 8.1032 | 18 | 0.9771 |
| Inosine to EDTA ratio | GD | MR Egger | 15.7591 | 22 | 0.8277 |
| Inosine to EDTA ratio | GD | Inverse variance weighted | 15.8471 | 23 | 0.8617 |
| Mannose to glycerol ratio | GD | MR Egger | 12.1257 | 17 | 0.7925 |
| Mannose to glycerol ratio | GD | Inverse variance weighted | 13.8763 | 18 | 0.7371 |
| Mannose to mannitol to sorbitol ratio | GD | MR Egger | 18.8514 | 19 | 0.4664 |
| Mannose to mannitol to sorbitol ratio | GD | Inverse variance weighted | 19.9792 | 20 | 0.4592 |
| Acetylcarnitine (C2) to propionylcarnitine (C3) ratio | GD | MR Egger | 12.2790 | 17 | 0.7830 |
| Acetylcarnitine (C2) to propionylcarnitine (C3) ratio | GD | Inverse variance weighted | 12.8155 | 18 | 0.8024 |
| Proline to trans-4-hydroxyproline ratio | GD | MR Egger | 9.2982 | 22 | 0.9916 |
| Proline to trans-4-hydroxyproline ratio | GD | Inverse variance weighted | 9.3140 | 23 | 0.9948 |
| Adenosine 5'-diphosphate (ADP) to ornithine ratio | GD | MR Egger | 16.1272 | 21 | 0.7625 |
| Adenosine 5'-diphosphate (ADP) to ornithine ratio | GD | Inverse variance weighted | 20.3870 | 22 | 0.5588 |
| Fructose to maltose ratio | GD | MR Egger | 20.9881 | 20 | 0.3978 |
| Fructose to maltose ratio | GD | Inverse variance weighted | 21.8410 | 21 | 0.4087 |

**TableS10 Directional horizontal pleiotropy assessed by intercept term in MR Egger regression of the association between metabolite and GD**

| **Exposure** | **Outcome** | **Egger intercept** | **SE** | **Pvalue** |
| --- | --- | --- | --- | --- |
| Glycerol 3-phosphate levels | GD | 0.0054 | 0.0199 | 0.7899 |
| 3-indoxyl sulfate levels | GD | 0.0389 | 0.0181 | 0.0451 |
| Docosapentaenoate (n6 DPA; 22:5n6) levels | GD | 0.0059 | 0.0340 | 0.8646 |
| 1-ribosyl-imidazoleacetate levels | GD | -0.0178 | 0.0192 | 0.3602 |
| Mannitol/sorbitol levels | GD | 0.0176 | 0.0200 | 0.3885 |
| Hexanoylglutamine levels | GD | 0.0068 | 0.0200 | 0.7366 |
| 4-allylphenol sulfate levels | GD | -0.0345 | 0.0173 | 0.0600 |
| 21-hydroxypregnenolone disulfate levels | GD | -0.0038 | 0.0163 | 0.8183 |
| 2,3-dihydroxyisovalerate levels | GD | -0.0065 | 0.0149 | 0.6676 |
| Isoursodeoxycholate levels | GD | -0.0081 | 0.0170 | 0.6401 |
| 2-stearoyl-GPE (18:0) levels | GD | 0.0098 | 0.0172 | 0.5742 |
| Dimethyl sulfone levels | GD | 0.0096 | 0.0205 | 0.6434 |
| S-allylcysteine levels | GD | 0.0104 | 0.0224 | 0.6508 |
| 4-methylguaiacol sulfate levels | GD | 0.0105 | 0.0269 | 0.7068 |
| 1-palmitoyl-2-stearoyl-gpc (16:0/18:0) levels | GD | -0.0346 | 0.0176 | 0.0597 |
| 1-(1-enyl-palmitoyl)-2-oleoyl-GPE (p-16:0/18:1) levels | GD | 0.0180 | 0.0185 | 0.3413 |
| Pimeloylcarnitine/3-methyladipoylcarnitine (C7-DC) levels | GD | 0.0164 | 0.0205 | 0.4323 |
| N-stearoyl-sphingadienine (d18:2/18:0) levels | GD | 0.0347 | 0.0217 | 0.1262 |
| Ethyl alpha-glucopyranoside levels | GD | 0.0433 | 0.0172 | 0.0183 |
| Glycosyl ceramide (d18:1/20:0, d16:1/22:0) levels | GD | -0.0039 | 0.0169 | 0.8173 |
| Cortolone glucuronide (1) levels | GD | 0.0156 | 0.0174 | 0.3810 |
| 5-hydroxy-2-methylpyridine sulfate levels | GD | -0.0138 | 0.0254 | 0.5945 |
| 4-acetylcatechol sulfate (1) levels | GD | -0.0027 | 0.0150 | 0.8578 |
| Pregnenetriol sulfate levels | GD | -0.0013 | 0.0097 | 0.8931 |
| 3-hydroxyoctanoylcarnitine (1) levels | GD | 0.0454 | 0.0257 | 0.0984 |
| Methyl indole-3-acetate levels | GD | 0.0395 | 0.0201 | 0.0647 |
| Gamma-glutamylglutamine levels | GD | -0.0078 | 0.0140 | 0.5812 |
| 2-hydroxystearate levels | GD | -0.0073 | 0.0214 | 0.7362 |
| 1-palmitoyl-2-linoleoyl-gpc (16:0/18:2) levels | GD | 0.0252 | 0.0145 | 0.0929 |
| Stearoyl sphingomyelin (d18:1/18:0) levels | GD | -0.0200 | 0.0162 | 0.2280 |
| Adenosine 5'-diphosphate (ADP) to fructose ratio | GD | 0.0193 | 0.0143 | 0.1897 |
| Adenosine 5'-diphosphate (ADP) to 2'-deoxyuridine ratio | GD | -0.0237 | 0.0202 | 0.2568 |
| Phosphate to alanine ratio | GD | 0.0368 | 0.0204 | 0.0906 |
| Spermidine to phosphate ratio | GD | -0.0256 | 0.0184 | 0.1823 |
| Adenosine 5'-diphosphate (ADP) to oxalate (ethanedioate) ratio | GD | 0.0095 | 0.0108 | 0.3918 |
| Cysteinylglycine to glutamate ratio | GD | -0.0010 | 0.0176 | 0.9543 |
| Adenosine 5'-monophosphate (AMP) to glutamate ratio | GD | -0.0209 | 0.0236 | 0.3850 |
| Citrate to 4-hydroxyphenylpyruvate ratio | GD | 0.0165 | 0.0223 | 0.4697 |
| Inosine to EDTA ratio | GD | -0.0060 | 0.0203 | 0.7695 |
| Mannose to glycerol ratio | GD | -0.0206 | 0.0155 | 0.2033 |
| Mannose to mannitol to sorbitol ratio | GD | 0.0291 | 0.0274 | 0.3016 |
| Acetylcarnitine (C2) to propionylcarnitine (C3) ratio | GD | 0.0148 | 0.0202 | 0.4739 |
| Proline to trans-4-hydroxyproline ratio | GD | 0.0030 | 0.0236 | 0.9011 |
| Adenosine 5'-diphosphate (ADP) to ornithine ratio | GD | 0.0399 | 0.0193 | 0.0516 |
| Fructose to maltose ratio | GD | 0.0202 | 0.0224 | 0.3781 |

**Table S11 Instrumental variables used in MR analysis of the association between gut microbiota and blood metabolites**

| **Exposure** | **Outcome** | **SNP** | **Exposure** | | | **Outcome** | | |
| --- | --- | --- | --- | --- | --- | --- | --- | --- |
|  |  |  | **BETA** | **SE** | **P** | **BETA** | **SE** | **P** |
| genus.Catenibacterium.id.2153 | Glycerol 3-phosphate | rs12404911 | 0.141 | 0.030 | 0.000 | -0.019 | 0.020 | 0.344 |
| genus.Catenibacterium.id.2153 | Glycerol 3-phosphate | rs212393 | -0.135 | 0.029 | 0.000 | -0.002 | 0.017 | 0.903 |
| genus.Catenibacterium.id.2153 | Glycerol 3-phosphate | rs73128290 | 0.130 | 0.028 | 0.000 | -0.029 | 0.018 | 0.096 |
| genus.Catenibacterium.id.2153 | Glycerol 3-phosphate | rs77285108 | -0.162 | 0.035 | 0.000 | 0.035 | 0.022 | 0.118 |
| genus.Catenibacterium.id.2153 | Glycerol 3-phosphate | rs7742829 | 0.114 | 0.025 | 0.000 | -0.010 | 0.016 | 0.553 |
| genus.Dialister.id.2183 | 21-hydroxypregnenolone disulfate l | rs10086221 | -0.062 | 0.013 | 0.000 | -0.016 | 0.015 | 0.273 |
| genus.Dialister.id.2183 | 21-hydroxypregnenolone disulfate l | rs10086225 | -0.062 | 0.013 | 0.000 | -0.016 | 0.015 | 0.270 |
| genus.Dialister.id.2183 | 21-hydroxypregnenolone disulfate l | rs10105942 | -0.061 | 0.013 | 0.000 | -0.017 | 0.015 | 0.245 |
| genus.Dialister.id.2183 | 21-hydroxypregnenolone disulfate l | rs10106300 | -0.062 | 0.013 | 0.000 | -0.017 | 0.015 | 0.251 |
| genus.Dialister.id.2183 | 21-hydroxypregnenolone disulfate l | rs10938938 | 0.077 | 0.017 | 0.000 | -0.028 | 0.019 | 0.142 |
| genus.Dialister.id.2183 | 21-hydroxypregnenolone disulfate l | rs11071887 | 0.066 | 0.015 | 0.000 | 0.011 | 0.017 | 0.499 |
| genus.Dialister.id.2183 | 21-hydroxypregnenolone disulfate l | rs11166701 | -0.066 | 0.013 | 0.000 | -0.034 | 0.015 | 0.019 |
| genus.Dialister.id.2183 | 21-hydroxypregnenolone disulfate l | rs12334717 | -0.059 | 0.013 | 0.000 | -0.034 | 0.015 | 0.020 |
| genus.Dialister.id.2183 | 21-hydroxypregnenolone disulfate l | rs1601589 | 0.101 | 0.023 | 0.000 | 0.028 | 0.025 | 0.259 |
| genus.Dialister.id.2183 | 21-hydroxypregnenolone disulfate l | rs17541540 | 0.059 | 0.013 | 0.000 | -0.021 | 0.015 | 0.148 |
| genus.Dialister.id.2183 | 21-hydroxypregnenolone disulfate l | rs2257073 | 0.065 | 0.015 | 0.000 | -0.005 | 0.016 | 0.778 |
| genus.Dialister.id.2183 | 21-hydroxypregnenolone disulfate l | rs2257090 | 0.066 | 0.015 | 0.000 | -0.002 | 0.016 | 0.894 |
| genus.Dialister.id.2183 | 21-hydroxypregnenolone disulfate l | rs2314294 | 0.087 | 0.019 | 0.000 | -0.008 | 0.021 | 0.692 |
| genus.Dialister.id.2183 | 21-hydroxypregnenolone disulfate l | rs2390568 | 0.073 | 0.016 | 0.000 | -0.019 | 0.018 | 0.305 |
| genus.Dialister.id.2183 | 21-hydroxypregnenolone disulfate l | rs2390569 | 0.073 | 0.016 | 0.000 | -0.017 | 0.018 | 0.336 |
| genus.Dialister.id.2183 | 21-hydroxypregnenolone disulfate l | rs2435610 | 0.065 | 0.014 | 0.000 | -0.007 | 0.016 | 0.650 |
| genus.Dialister.id.2183 | 21-hydroxypregnenolone disulfate l | rs34929978 | -0.057 | 0.013 | 0.000 | 0.009 | 0.015 | 0.561 |
| genus.Dialister.id.2183 | 21-hydroxypregnenolone disulfate l | rs4263802 | -0.063 | 0.013 | 0.000 | -0.016 | 0.015 | 0.287 |
| genus.Dialister.id.2183 | 21-hydroxypregnenolone disulfate l | rs4355808 | -0.063 | 0.013 | 0.000 | -0.016 | 0.015 | 0.287 |
| genus.Dialister.id.2183 | 21-hydroxypregnenolone disulfate l | rs4725397 | 0.065 | 0.015 | 0.000 | -0.006 | 0.016 | 0.727 |
| genus.Dialister.id.2183 | 21-hydroxypregnenolone disulfate l | rs4747450 | 0.067 | 0.015 | 0.000 | 0.007 | 0.017 | 0.666 |
| genus.Dialister.id.2183 | 21-hydroxypregnenolone disulfate l | rs4753063 | 0.060 | 0.013 | 0.000 | 0.002 | 0.015 | 0.897 |
| genus.Dialister.id.2183 | 21-hydroxypregnenolone disulfate l | rs4909362 | -0.063 | 0.013 | 0.000 | -0.016 | 0.015 | 0.278 |
| genus.Dialister.id.2183 | 21-hydroxypregnenolone disulfate l | rs517089 | 0.076 | 0.017 | 0.000 | 0.015 | 0.020 | 0.434 |
| genus.Dialister.id.2183 | 21-hydroxypregnenolone disulfate l | rs57053627 | 0.065 | 0.015 | 0.000 | 0.009 | 0.017 | 0.608 |
| genus.Dialister.id.2183 | 21-hydroxypregnenolone disulfate l | rs6982344 | -0.064 | 0.013 | 0.000 | -0.017 | 0.015 | 0.246 |
| genus.Dialister.id.2183 | 21-hydroxypregnenolone disulfate l | rs75416973 | 0.073 | 0.016 | 0.000 | -0.005 | 0.020 | 0.795 |
| genus.Dialister.id.2183 | 21-hydroxypregnenolone disulfate l | rs764177 | -0.060 | 0.014 | 0.000 | 0.002 | 0.016 | 0.877 |
| genus.Dialister.id.2183 | 21-hydroxypregnenolone disulfate l | rs76680460 | -0.161 | 0.036 | 0.000 | -0.069 | 0.033 | 0.035 |
| genus.Dialister.id.2183 | 21-hydroxypregnenolone disulfate l | rs7814375 | -0.064 | 0.013 | 0.000 | -0.017 | 0.015 | 0.249 |
| genus.Dialister.id.2183 | 21-hydroxypregnenolone disulfate l | rs7816464 | -0.060 | 0.013 | 0.000 | -0.016 | 0.015 | 0.271 |
| genus.Dialister.id.2183 | 21-hydroxypregnenolone disulfate l | rs7829832 | -0.061 | 0.013 | 0.000 | -0.016 | 0.015 | 0.282 |
| genus.Dialister.id.2183 | 21-hydroxypregnenolone disulfate l | rs9554744 | 0.073 | 0.016 | 0.000 | -0.018 | 0.018 | 0.324 |
| genus.Dialister.id.2183 | 21-hydroxypregnenolone disulfate l | rs9557564 | 0.073 | 0.016 | 0.000 | -0.018 | 0.018 | 0.324 |
| genus.Dialister.id.2183 | Inosine to EDTA ratio | rs10086221 | -0.062 | 0.013 | 0.000 | -0.034 | 0.020 | 0.096 |
| genus.Dialister.id.2183 | Inosine to EDTA ratio | rs10086225 | -0.062 | 0.013 | 0.000 | -0.034 | 0.020 | 0.095 |
| genus.Dialister.id.2183 | Inosine to EDTA ratio | rs10105942 | -0.061 | 0.013 | 0.000 | -0.035 | 0.020 | 0.084 |
| genus.Dialister.id.2183 | Inosine to EDTA ratio | rs10106300 | -0.062 | 0.013 | 0.000 | -0.035 | 0.020 | 0.086 |
| genus.Dialister.id.2183 | Inosine to EDTA ratio | rs10938938 | 0.077 | 0.017 | 0.000 | -0.039 | 0.025 | 0.122 |
| genus.Dialister.id.2183 | Inosine to EDTA ratio | rs11071887 | 0.066 | 0.015 | 0.000 | -0.005 | 0.022 | 0.837 |
| genus.Dialister.id.2183 | Inosine to EDTA ratio | rs11166701 | -0.066 | 0.013 | 0.000 | -0.030 | 0.020 | 0.125 |
| genus.Dialister.id.2183 | Inosine to EDTA ratio | rs12334717 | -0.059 | 0.013 | 0.000 | -0.024 | 0.020 | 0.224 |
| genus.Dialister.id.2183 | Inosine to EDTA ratio | rs1601589 | 0.101 | 0.023 | 0.000 | -0.001 | 0.034 | 0.977 |
| genus.Dialister.id.2183 | Inosine to EDTA ratio | rs17541540 | 0.059 | 0.013 | 0.000 | -0.005 | 0.020 | 0.789 |
| genus.Dialister.id.2183 | Inosine to EDTA ratio | rs2257073 | 0.065 | 0.015 | 0.000 | -0.031 | 0.022 | 0.164 |
| genus.Dialister.id.2183 | Inosine to EDTA ratio | rs2257090 | 0.066 | 0.015 | 0.000 | -0.034 | 0.022 | 0.126 |
| genus.Dialister.id.2183 | Inosine to EDTA ratio | rs2314294 | 0.087 | 0.019 | 0.000 | 0.006 | 0.029 | 0.833 |
| genus.Dialister.id.2183 | Inosine to EDTA ratio | rs2390568 | 0.073 | 0.016 | 0.000 | 0.010 | 0.025 | 0.681 |
| genus.Dialister.id.2183 | Inosine to EDTA ratio | rs2390569 | 0.073 | 0.016 | 0.000 | 0.009 | 0.025 | 0.710 |
| genus.Dialister.id.2183 | Inosine to EDTA ratio | rs2435610 | 0.065 | 0.014 | 0.000 | -0.031 | 0.022 | 0.165 |
| genus.Dialister.id.2183 | Inosine to EDTA ratio | rs34929978 | -0.057 | 0.013 | 0.000 | -0.010 | 0.020 | 0.639 |
| genus.Dialister.id.2183 | Inosine to EDTA ratio | rs4263802 | -0.063 | 0.013 | 0.000 | -0.034 | 0.020 | 0.090 |
| genus.Dialister.id.2183 | Inosine to EDTA ratio | rs4355808 | -0.063 | 0.013 | 0.000 | -0.034 | 0.020 | 0.096 |
| genus.Dialister.id.2183 | Inosine to EDTA ratio | rs4725397 | 0.065 | 0.015 | 0.000 | -0.031 | 0.022 | 0.167 |
| genus.Dialister.id.2183 | Inosine to EDTA ratio | rs4747450 | 0.067 | 0.015 | 0.000 | 0.011 | 0.023 | 0.624 |
| genus.Dialister.id.2183 | Inosine to EDTA ratio | rs4753063 | 0.060 | 0.013 | 0.000 | 0.031 | 0.020 | 0.126 |
| genus.Dialister.id.2183 | Inosine to EDTA ratio | rs4909362 | -0.063 | 0.013 | 0.000 | -0.034 | 0.020 | 0.096 |
| genus.Dialister.id.2183 | Inosine to EDTA ratio | rs517089 | 0.076 | 0.017 | 0.000 | -0.001 | 0.027 | 0.983 |
| genus.Dialister.id.2183 | Inosine to EDTA ratio | rs57053627 | 0.065 | 0.015 | 0.000 | 0.011 | 0.023 | 0.632 |
| genus.Dialister.id.2183 | Inosine to EDTA ratio | rs6982344 | -0.064 | 0.013 | 0.000 | -0.035 | 0.020 | 0.083 |
| genus.Dialister.id.2183 | Inosine to EDTA ratio | rs75416973 | 0.073 | 0.016 | 0.000 | 0.024 | 0.027 | 0.376 |
| genus.Dialister.id.2183 | Inosine to EDTA ratio | rs764177 | -0.060 | 0.014 | 0.000 | 0.030 | 0.021 | 0.163 |
| genus.Dialister.id.2183 | Inosine to EDTA ratio | rs76680460 | -0.161 | 0.036 | 0.000 | -0.003 | 0.044 | 0.942 |
| genus.Dialister.id.2183 | Inosine to EDTA ratio | rs7814375 | -0.064 | 0.013 | 0.000 | -0.035 | 0.020 | 0.085 |
| genus.Dialister.id.2183 | Inosine to EDTA ratio | rs7816464 | -0.060 | 0.013 | 0.000 | -0.034 | 0.020 | 0.087 |
| genus.Dialister.id.2183 | Inosine to EDTA ratio | rs7829832 | -0.061 | 0.013 | 0.000 | -0.035 | 0.020 | 0.082 |
| genus.Dialister.id.2183 | Inosine to EDTA ratio | rs9554744 | 0.073 | 0.016 | 0.000 | 0.009 | 0.025 | 0.731 |
| genus.Dialister.id.2183 | Inosine to EDTA ratio | rs9557564 | 0.073 | 0.016 | 0.000 | 0.009 | 0.025 | 0.712 |
| genus.Dialister.id.2183 | Mannose to glycerol ratio | rs10086221 | -0.062 | 0.013 | 0.000 | 0.008 | 0.014 | 0.547 |
| genus.Dialister.id.2183 | Mannose to glycerol ratio | rs10086225 | -0.062 | 0.013 | 0.000 | 0.009 | 0.014 | 0.531 |
| genus.Dialister.id.2183 | Mannose to glycerol ratio | rs10105942 | -0.061 | 0.013 | 0.000 | 0.009 | 0.014 | 0.508 |
| genus.Dialister.id.2183 | Mannose to glycerol ratio | rs10106300 | -0.062 | 0.013 | 0.000 | 0.010 | 0.014 | 0.485 |
| genus.Dialister.id.2183 | Mannose to glycerol ratio | rs10938938 | 0.077 | 0.017 | 0.000 | 0.028 | 0.018 | 0.108 |
| genus.Dialister.id.2183 | Mannose to glycerol ratio | rs11071887 | 0.066 | 0.015 | 0.000 | 0.027 | 0.015 | 0.074 |
| genus.Dialister.id.2183 | Mannose to glycerol ratio | rs11166701 | -0.066 | 0.013 | 0.000 | 0.011 | 0.013 | 0.432 |
| genus.Dialister.id.2183 | Mannose to glycerol ratio | rs12334717 | -0.059 | 0.013 | 0.000 | 0.011 | 0.013 | 0.435 |
| genus.Dialister.id.2183 | Mannose to glycerol ratio | rs1601589 | 0.101 | 0.023 | 0.000 | 0.021 | 0.023 | 0.368 |
| genus.Dialister.id.2183 | Mannose to glycerol ratio | rs17541540 | 0.059 | 0.013 | 0.000 | 0.009 | 0.014 | 0.529 |
| genus.Dialister.id.2183 | Mannose to glycerol ratio | rs2257073 | 0.065 | 0.015 | 0.000 | -0.008 | 0.015 | 0.597 |
| genus.Dialister.id.2183 | Mannose to glycerol ratio | rs2257090 | 0.066 | 0.015 | 0.000 | -0.009 | 0.015 | 0.550 |
| genus.Dialister.id.2183 | Mannose to glycerol ratio | rs2314294 | 0.087 | 0.019 | 0.000 | 0.023 | 0.020 | 0.240 |
| genus.Dialister.id.2183 | Mannose to glycerol ratio | rs2390568 | 0.073 | 0.016 | 0.000 | -0.008 | 0.017 | 0.621 |
| genus.Dialister.id.2183 | Mannose to glycerol ratio | rs2390569 | 0.073 | 0.016 | 0.000 | -0.007 | 0.017 | 0.676 |
| genus.Dialister.id.2183 | Mannose to glycerol ratio | rs2435610 | 0.065 | 0.014 | 0.000 | -0.020 | 0.015 | 0.185 |
| genus.Dialister.id.2183 | Mannose to glycerol ratio | rs34929978 | -0.057 | 0.013 | 0.000 | 0.007 | 0.014 | 0.621 |
| genus.Dialister.id.2183 | Mannose to glycerol ratio | rs4263802 | -0.063 | 0.013 | 0.000 | 0.009 | 0.014 | 0.522 |
| genus.Dialister.id.2183 | Mannose to glycerol ratio | rs4355808 | -0.063 | 0.013 | 0.000 | 0.009 | 0.014 | 0.532 |
| genus.Dialister.id.2183 | Mannose to glycerol ratio | rs4725397 | 0.065 | 0.015 | 0.000 | -0.009 | 0.015 | 0.558 |
| genus.Dialister.id.2183 | Mannose to glycerol ratio | rs4747450 | 0.067 | 0.015 | 0.000 | 0.010 | 0.015 | 0.496 |
| genus.Dialister.id.2183 | Mannose to glycerol ratio | rs4753063 | 0.060 | 0.013 | 0.000 | -0.008 | 0.014 | 0.542 |
| genus.Dialister.id.2183 | Mannose to glycerol ratio | rs4909362 | -0.063 | 0.013 | 0.000 | 0.008 | 0.014 | 0.538 |
| genus.Dialister.id.2183 | Mannose to glycerol ratio | rs517089 | 0.076 | 0.017 | 0.000 | 0.032 | 0.018 | 0.080 |
| genus.Dialister.id.2183 | Mannose to glycerol ratio | rs57053627 | 0.065 | 0.015 | 0.000 | 0.010 | 0.015 | 0.518 |
| genus.Dialister.id.2183 | Mannose to glycerol ratio | rs6982344 | -0.064 | 0.013 | 0.000 | 0.011 | 0.014 | 0.439 |
| genus.Dialister.id.2183 | Mannose to glycerol ratio | rs75416973 | 0.073 | 0.016 | 0.000 | -0.010 | 0.018 | 0.594 |
| genus.Dialister.id.2183 | Mannose to glycerol ratio | rs764177 | -0.060 | 0.014 | 0.000 | 0.008 | 0.014 | 0.584 |
| genus.Dialister.id.2183 | Mannose to glycerol ratio | rs76680460 | -0.161 | 0.036 | 0.000 | -0.008 | 0.030 | 0.788 |
| genus.Dialister.id.2183 | Mannose to glycerol ratio | rs7814375 | -0.064 | 0.013 | 0.000 | 0.011 | 0.014 | 0.438 |
| genus.Dialister.id.2183 | Mannose to glycerol ratio | rs7816464 | -0.060 | 0.013 | 0.000 | 0.009 | 0.014 | 0.519 |
| genus.Dialister.id.2183 | Mannose to glycerol ratio | rs7829832 | -0.061 | 0.013 | 0.000 | 0.008 | 0.014 | 0.582 |
| genus.Dialister.id.2183 | Mannose to glycerol ratio | rs9554744 | 0.073 | 0.016 | 0.000 | -0.006 | 0.017 | 0.708 |
| genus.Dialister.id.2183 | Mannose to glycerol ratio | rs9557564 | 0.073 | 0.016 | 0.000 | -0.008 | 0.017 | 0.615 |
| genus.Oscillospira.id.2064 | Adenosine 5'-diphosphate (ADP) to fructose ratio | rs11541674 | -0.078 | 0.017 | 0.000 | -0.008 | 0.025 | 0.755 |
| genus.Oscillospira.id.2064 | Adenosine 5'-diphosphate (ADP) to fructose ratio | rs12206468 | -0.133 | 0.027 | 0.000 | 0.083 | 0.042 | 0.047 |
| genus.Oscillospira.id.2064 | Adenosine 5'-diphosphate (ADP) to fructose ratio | rs12925026 | 0.136 | 0.031 | 0.000 | -0.033 | 0.039 | 0.401 |
| genus.Oscillospira.id.2064 | Adenosine 5'-diphosphate (ADP) to fructose ratio | rs1954532 | -0.083 | 0.018 | 0.000 | -0.007 | 0.026 | 0.797 |
| genus.Oscillospira.id.2064 | Adenosine 5'-diphosphate (ADP) to fructose ratio | rs28889936 | 0.114 | 0.025 | 0.000 | -0.063 | 0.040 | 0.114 |
| genus.Oscillospira.id.2064 | Adenosine 5'-diphosphate (ADP) to fructose ratio | rs62422654 | 0.090 | 0.020 | 0.000 | -0.009 | 0.029 | 0.746 |
| genus.Oscillospira.id.2064 | Adenosine 5'-diphosphate (ADP) to fructose ratio | rs72866977 | -0.131 | 0.028 | 0.000 | 0.002 | 0.041 | 0.957 |
| genus.Oscillospira.id.2064 | Adenosine 5'-diphosphate (ADP) to fructose ratio | rs73038677 | -0.083 | 0.017 | 0.000 | -0.004 | 0.027 | 0.888 |
| genus.Oscillospira.id.2064 | Adenosine 5'-diphosphate (ADP) to fructose ratio | rs751183 | -0.077 | 0.017 | 0.000 | 0.020 | 0.026 | 0.446 |
| genus.Oscillospira.id.2064 | Adenosine 5'-diphosphate (ADP) to fructose ratio | rs8076323 | 0.072 | 0.016 | 0.000 | -0.043 | 0.024 | 0.067 |
| genus.Oscillospira.id.2064 | Gamma-glutamylthreonine | rs11541674 | -0.078 | 0.017 | 0.000 | 0.018 | 0.019 | 0.336 |
| genus.Oscillospira.id.2064 | Gamma-glutamylthreonine | rs12206468 | -0.133 | 0.027 | 0.000 | 0.018 | 0.031 | 0.563 |
| genus.Oscillospira.id.2064 | Gamma-glutamylthreonine | rs12925026 | 0.136 | 0.031 | 0.000 | -0.028 | 0.030 | 0.339 |
| genus.Oscillospira.id.2064 | Gamma-glutamylthreonine | rs1954532 | 0.083 | 0.018 | 0.000 | -0.029 | 0.020 | 0.146 |
| genus.Oscillospira.id.2064 | Gamma-glutamylthreonine | rs28889936 | 0.114 | 0.025 | 0.000 | -0.020 | 0.029 | 0.490 |
| genus.Oscillospira.id.2064 | Gamma-glutamylthreonine | rs62422654 | 0.090 | 0.020 | 0.000 | -0.030 | 0.022 | 0.166 |
| genus.Oscillospira.id.2064 | Gamma-glutamylthreonine | rs72866977 | -0.131 | 0.028 | 0.000 | 0.050 | 0.030 | 0.099 |
| genus.Oscillospira.id.2064 | Gamma-glutamylthreonine | rs73038677 | -0.083 | 0.017 | 0.000 | -0.022 | 0.020 | 0.262 |
| genus.Oscillospira.id.2064 | Gamma-glutamylthreonine | rs751183 | 0.077 | 0.017 | 0.000 | 0.026 | 0.019 | 0.176 |
| genus.Oscillospira.id.2064 | Gamma-glutamylthreonine | rs8076323 | 0.072 | 0.016 | 0.000 | -0.011 | 0.018 | 0.540 |
| genus.Ruminiclostridium5.id.11355 | 1-(1-enyl-palmitoyl)-2-oleoyl-GPE (p-160181) levels | rs10827477 | -0.055 | 0.012 | 0.000 | -0.016 | 0.017 | 0.328 |
| genus.Ruminiclostridium5.id.11355 | 1-(1-enyl-palmitoyl)-2-oleoyl-GPE (p-160181) levels | rs113753996 | 0.082 | 0.017 | 0.000 | 0.001 | 0.026 | 0.966 |
| genus.Ruminiclostridium5.id.11355 | 1-(1-enyl-palmitoyl)-2-oleoyl-GPE (p-160181) levels | rs1223978 | 0.048 | 0.011 | 0.000 | 0.014 | 0.016 | 0.364 |
| genus.Ruminiclostridium5.id.11355 | 1-(1-enyl-palmitoyl)-2-oleoyl-GPE (p-160181) levels | rs1492620 | -0.083 | 0.018 | 0.000 | -0.010 | 0.027 | 0.701 |
| genus.Ruminiclostridium5.id.11355 | 1-(1-enyl-palmitoyl)-2-oleoyl-GPE (p-160181) levels | rs2286384 | -0.052 | 0.011 | 0.000 | 0.009 | 0.015 | 0.537 |
| genus.Ruminiclostridium5.id.11355 | 1-(1-enyl-palmitoyl)-2-oleoyl-GPE (p-160181) levels | rs2791343 | 0.052 | 0.011 | 0.000 | 0.002 | 0.016 | 0.893 |
| genus.Ruminiclostridium5.id.11355 | 1-(1-enyl-palmitoyl)-2-oleoyl-GPE (p-160181) levels | rs2801960 | 0.052 | 0.012 | 0.000 | -0.010 | 0.017 | 0.579 |
| genus.Ruminiclostridium5.id.11355 | 1-(1-enyl-palmitoyl)-2-oleoyl-GPE (p-160181) levels | rs2833828 | 0.049 | 0.011 | 0.000 | 0.017 | 0.016 | 0.274 |
| genus.Ruminiclostridium5.id.11355 | 1-(1-enyl-palmitoyl)-2-oleoyl-GPE (p-160181) levels | rs4955951 | -0.071 | 0.017 | 0.000 | 0.020 | 0.025 | 0.425 |
| genus.Ruminiclostridium5.id.11355 | 1-(1-enyl-palmitoyl)-2-oleoyl-GPE (p-160181) levels | rs6121460 | 0.093 | 0.020 | 0.000 | -0.003 | 0.030 | 0.929 |
| genus.Ruminiclostridium5.id.11355 | 1-(1-enyl-palmitoyl)-2-oleoyl-GPE (p-160181) levels | rs73002572 | 0.182 | 0.041 | 0.000 | 0.115 | 0.043 | 0.007 |
| genus.Ruminiclostridium5.id.11355 | 1-(1-enyl-palmitoyl)-2-oleoyl-GPE (p-160181) levels | rs79968837 | -0.095 | 0.019 | 0.000 | -0.019 | 0.029 | 0.500 |
| genus.Ruminiclostridium5.id.11355 | 1-(1-enyl-palmitoyl)-2-oleoyl-GPE (p-160181) levels | rs8053158 | -0.074 | 0.016 | 0.000 | -0.040 | 0.024 | 0.097 |
| order.MollicutesRF9.id.11579 | Adenosine 5'-diphosphate (ADP) to fructose ratio | rs10071529 | -0.125 | 0.028 | 0.000 | -0.004 | 0.041 | 0.931 |
| order.MollicutesRF9.id.11579 | Adenosine 5'-diphosphate (ADP) to fructose ratio | rs114184530 | -0.155 | 0.034 | 0.000 | 0.085 | 0.049 | 0.079 |
| order.MollicutesRF9.id.11579 | Adenosine 5'-diphosphate (ADP) to fructose ratio | rs11779863 | -0.077 | 0.017 | 0.000 | 0.007 | 0.025 | 0.789 |
| order.MollicutesRF9.id.11579 | Adenosine 5'-diphosphate (ADP) to fructose ratio | rs13100746 | 0.064 | 0.014 | 0.000 | -0.049 | 0.021 | 0.021 |
| order.MollicutesRF9.id.11579 | Adenosine 5'-diphosphate (ADP) to fructose ratio | rs17235252 | -0.122 | 0.026 | 0.000 | -0.030 | 0.035 | 0.388 |
| order.MollicutesRF9.id.11579 | Adenosine 5'-diphosphate (ADP) to fructose ratio | rs3932485 | 0.063 | 0.014 | 0.000 | -0.025 | 0.021 | 0.237 |
| order.MollicutesRF9.id.11579 | Adenosine 5'-diphosphate (ADP) to fructose ratio | rs515984 | 0.088 | 0.019 | 0.000 | -0.037 | 0.029 | 0.203 |
| order.MollicutesRF9.id.11579 | Adenosine 5'-diphosphate (ADP) to fructose ratio | rs62188991 | -0.111 | 0.024 | 0.000 | 0.043 | 0.038 | 0.258 |
| order.MollicutesRF9.id.11579 | Adenosine 5'-diphosphate (ADP) to fructose ratio | rs638542 | 0.071 | 0.016 | 0.000 | -0.015 | 0.023 | 0.528 |
| order.MollicutesRF9.id.11579 | Adenosine 5'-diphosphate (ADP) to fructose ratio | rs739151 | -0.065 | 0.014 | 0.000 | -0.019 | 0.021 | 0.360 |
| order.MollicutesRF9.id.11579 | Adenosine 5'-diphosphate (ADP) to fructose ratio | rs74603314 | 0.231 | 0.049 | 0.000 | -0.045 | 0.047 | 0.333 |
| order.MollicutesRF9.id.11579 | Adenosine 5'-diphosphate (ADP) to fructose ratio | rs76373661 | 0.091 | 0.020 | 0.000 | -0.068 | 0.031 | 0.027 |
| order.MollicutesRF9.id.11579 | Adenosine 5'-diphosphate (ADP) to fructose ratio | rs7706512 | -0.066 | 0.014 | 0.000 | 0.022 | 0.021 | 0.293 |
| order.MollicutesRF9.id.11579 | Adenosine 5'-diphosphate (ADP) to fructose ratio | rs7801843 | -0.087 | 0.019 | 0.000 | -0.028 | 0.030 | 0.352 |
| order.MollicutesRF9.id.11579 | Adenosine 5'-diphosphate (ADP) to fructose ratio | rs949341 | -0.066 | 0.015 | 0.000 | -0.014 | 0.022 | 0.513 |
| order.MollicutesRF9.id.11579 | Mannose to mannitol to sorbitol ratio | rs10071529 | -0.125 | 0.028 | 0.000 | 0.006 | 0.030 | 0.844 |
| order.MollicutesRF9.id.11579 | Mannose to mannitol to sorbitol ratio | rs114184530 | -0.155 | 0.034 | 0.000 | 0.001 | 0.036 | 0.972 |
| order.MollicutesRF9.id.11579 | Mannose to mannitol to sorbitol ratio | rs11779863 | -0.077 | 0.017 | 0.000 | 0.055 | 0.019 | 0.003 |
| order.MollicutesRF9.id.11579 | Mannose to mannitol to sorbitol ratio | rs13100746 | 0.064 | 0.014 | 0.000 | -0.007 | 0.016 | 0.660 |
| order.MollicutesRF9.id.11579 | Mannose to mannitol to sorbitol ratio | rs17235252 | -0.122 | 0.026 | 0.000 | 0.017 | 0.027 | 0.522 |
| order.MollicutesRF9.id.11579 | Mannose to mannitol to sorbitol ratio | rs3932485 | 0.063 | 0.014 | 0.000 | 0.004 | 0.016 | 0.819 |
| order.MollicutesRF9.id.11579 | Mannose to mannitol to sorbitol ratio | rs515984 | 0.088 | 0.019 | 0.000 | -0.028 | 0.022 | 0.197 |
| order.MollicutesRF9.id.11579 | Mannose to mannitol to sorbitol ratio | rs62188991 | -0.111 | 0.024 | 0.000 | -0.020 | 0.028 | 0.471 |
| order.MollicutesRF9.id.11579 | Mannose to mannitol to sorbitol ratio | rs638542 | 0.071 | 0.016 | 0.000 | 0.026 | 0.017 | 0.140 |
| order.MollicutesRF9.id.11579 | Mannose to mannitol to sorbitol ratio | rs739151 | -0.065 | 0.014 | 0.000 | -0.008 | 0.016 | 0.621 |
| order.MollicutesRF9.id.11579 | Mannose to mannitol to sorbitol ratio | rs74603314 | 0.231 | 0.049 | 0.000 | -0.042 | 0.034 | 0.223 |
| order.MollicutesRF9.id.11579 | Mannose to mannitol to sorbitol ratio | rs76373661 | 0.091 | 0.020 | 0.000 | 0.009 | 0.023 | 0.693 |
| order.MollicutesRF9.id.11579 | Mannose to mannitol to sorbitol ratio | rs7706512 | -0.066 | 0.014 | 0.000 | 0.019 | 0.016 | 0.215 |
| order.MollicutesRF9.id.11579 | Mannose to mannitol to sorbitol ratio | rs7801843 | -0.087 | 0.019 | 0.000 | 0.035 | 0.022 | 0.109 |
| order.MollicutesRF9.id.11579 | Mannose to mannitol to sorbitol ratio | rs949341 | -0.066 | 0.015 | 0.000 | 0.001 | 0.017 | 0.942 |
| order.MollicutesRF9.id.11579 | 2-hydroxystearate | rs10071529 | -0.125 | 0.028 | 0.000 | 0.030 | 0.030 | 0.319 |
| order.MollicutesRF9.id.11579 | 2-hydroxystearate | rs114184530 | -0.155 | 0.034 | 0.000 | 0.006 | 0.036 | 0.876 |
| order.MollicutesRF9.id.11579 | 2-hydroxystearate | rs11779863 | -0.077 | 0.017 | 0.000 | 0.012 | 0.018 | 0.523 |
| order.MollicutesRF9.id.11579 | 2-hydroxystearate | rs13100746 | 0.064 | 0.014 | 0.000 | -0.016 | 0.016 | 0.313 |
| order.MollicutesRF9.id.11579 | 2-hydroxystearate | rs17235252 | -0.122 | 0.026 | 0.000 | 0.036 | 0.026 | 0.177 |
| order.MollicutesRF9.id.11579 | 2-hydroxystearate | rs3932485 | 0.063 | 0.014 | 0.000 | 0.010 | 0.016 | 0.512 |
| order.MollicutesRF9.id.11579 | 2-hydroxystearate | rs515984 | 0.088 | 0.019 | 0.000 | 0.016 | 0.021 | 0.458 |
| order.MollicutesRF9.id.11579 | 2-hydroxystearate | rs62188991 | -0.111 | 0.024 | 0.000 | -0.035 | 0.028 | 0.211 |
| order.MollicutesRF9.id.11579 | 2-hydroxystearate | rs638542 | 0.071 | 0.016 | 0.000 | -0.020 | 0.017 | 0.261 |
| order.MollicutesRF9.id.11579 | 2-hydroxystearate | rs739151 | -0.065 | 0.014 | 0.000 | -0.005 | 0.015 | 0.750 |
| order.MollicutesRF9.id.11579 | 2-hydroxystearate | rs74603314 | 0.231 | 0.049 | 0.000 | -0.052 | 0.034 | 0.129 |
| order.MollicutesRF9.id.11579 | 2-hydroxystearate | rs76373661 | 0.091 | 0.020 | 0.000 | -0.012 | 0.023 | 0.580 |
| order.MollicutesRF9.id.11579 | 2-hydroxystearate | rs7706512 | -0.066 | 0.014 | 0.000 | -0.002 | 0.015 | 0.911 |
| order.MollicutesRF9.id.11579 | 2-hydroxystearate | rs7801843 | -0.087 | 0.019 | 0.000 | -0.007 | 0.022 | 0.748 |
| order.MollicutesRF9.id.11579 | 2-hydroxystearate | rs949341 | -0.066 | 0.015 | 0.000 | 0.029 | 0.016 | 0.081 |
| order.MollicutesRF9.id.11579 | Acetylcarnitine (C2) to propionylcarnitine (C3) ratio | rs10071529 | -0.125 | 0.028 | 0.000 | 0.025 | 0.029 | 0.395 |
| order.MollicutesRF9.id.11579 | Acetylcarnitine (C2) to propionylcarnitine (C3) ratio | rs114184530 | -0.155 | 0.034 | 0.000 | 0.022 | 0.035 | 0.523 |
| order.MollicutesRF9.id.11579 | Acetylcarnitine (C2) to propionylcarnitine (C3) ratio | rs11779863 | -0.077 | 0.017 | 0.000 | 0.031 | 0.018 | 0.084 |
| order.MollicutesRF9.id.11579 | Acetylcarnitine (C2) to propionylcarnitine (C3) ratio | rs13100746 | 0.064 | 0.014 | 0.000 | -0.011 | 0.015 | 0.476 |
| order.MollicutesRF9.id.11579 | Acetylcarnitine (C2) to propionylcarnitine (C3) ratio | rs17235252 | -0.122 | 0.026 | 0.000 | 0.000 | 0.026 | 0.990 |
| order.MollicutesRF9.id.11579 | Acetylcarnitine (C2) to propionylcarnitine (C3) ratio | rs3932485 | 0.063 | 0.014 | 0.000 | 0.001 | 0.015 | 0.935 |
| order.MollicutesRF9.id.11579 | Acetylcarnitine (C2) to propionylcarnitine (C3) ratio | rs515984 | 0.088 | 0.019 | 0.000 | -0.027 | 0.021 | 0.199 |
| order.MollicutesRF9.id.11579 | Acetylcarnitine (C2) to propionylcarnitine (C3) ratio | rs62188991 | -0.111 | 0.024 | 0.000 | 0.014 | 0.027 | 0.596 |
| order.MollicutesRF9.id.11579 | Acetylcarnitine (C2) to propionylcarnitine (C3) ratio | rs638542 | 0.071 | 0.016 | 0.000 | -0.032 | 0.017 | 0.061 |
| order.MollicutesRF9.id.11579 | Acetylcarnitine (C2) to propionylcarnitine (C3) ratio | rs739151 | -0.065 | 0.014 | 0.000 | -0.030 | 0.015 | 0.045 |
| order.MollicutesRF9.id.11579 | Acetylcarnitine (C2) to propionylcarnitine (C3) ratio | rs74603314 | 0.231 | 0.049 | 0.000 | -0.022 | 0.033 | 0.512 |
| order.MollicutesRF9.id.11579 | Acetylcarnitine (C2) to propionylcarnitine (C3) ratio | rs76373661 | 0.091 | 0.020 | 0.000 | -0.045 | 0.022 | 0.041 |
| order.MollicutesRF9.id.11579 | Acetylcarnitine (C2) to propionylcarnitine (C3) ratio | rs7706512 | -0.066 | 0.014 | 0.000 | -0.001 | 0.015 | 0.930 |
| order.MollicutesRF9.id.11579 | Acetylcarnitine (C2) to propionylcarnitine (C3) ratio | rs7801843 | -0.087 | 0.019 | 0.000 | 0.026 | 0.021 | 0.230 |
| order.MollicutesRF9.id.11579 | Acetylcarnitine (C2) to propionylcarnitine (C3) ratio | rs949341 | -0.066 | 0.015 | 0.000 | -0.002 | 0.016 | 0.897 |
| order.MollicutesRF9.id.11579 | Adenosine 5'-diphosphate (ADP) to ornithine ratio | rs10071529 | -0.125 | 0.028 | 0.000 | -0.029 | 0.041 | 0.482 |
| order.MollicutesRF9.id.11579 | Adenosine 5'-diphosphate (ADP) to ornithine ratio | rs114184530 | -0.155 | 0.034 | 0.000 | 0.016 | 0.049 | 0.737 |
| order.MollicutesRF9.id.11579 | Adenosine 5'-diphosphate (ADP) to ornithine ratio | rs11779863 | -0.077 | 0.017 | 0.000 | 0.014 | 0.025 | 0.587 |
| order.MollicutesRF9.id.11579 | Adenosine 5'-diphosphate (ADP) to ornithine ratio | rs13100746 | 0.064 | 0.014 | 0.000 | -0.018 | 0.021 | 0.392 |
| order.MollicutesRF9.id.11579 | Adenosine 5'-diphosphate (ADP) to ornithine ratio | rs17235252 | -0.122 | 0.026 | 0.000 | 0.023 | 0.035 | 0.514 |
| order.MollicutesRF9.id.11579 | Adenosine 5'-diphosphate (ADP) to ornithine ratio | rs3932485 | 0.063 | 0.014 | 0.000 | -0.005 | 0.021 | 0.800 |
| order.MollicutesRF9.id.11579 | Adenosine 5'-diphosphate (ADP) to ornithine ratio | rs515984 | 0.088 | 0.019 | 0.000 | -0.045 | 0.029 | 0.121 |
| order.MollicutesRF9.id.11579 | Adenosine 5'-diphosphate (ADP) to ornithine ratio | rs62188991 | -0.111 | 0.024 | 0.000 | 0.012 | 0.038 | 0.760 |
| order.MollicutesRF9.id.11579 | Adenosine 5'-diphosphate (ADP) to ornithine ratio | rs638542 | 0.071 | 0.016 | 0.000 | -0.013 | 0.023 | 0.566 |
| order.MollicutesRF9.id.11579 | Adenosine 5'-diphosphate (ADP) to ornithine ratio | rs739151 | -0.065 | 0.014 | 0.000 | -0.001 | 0.021 | 0.973 |
| order.MollicutesRF9.id.11579 | Adenosine 5'-diphosphate (ADP) to ornithine ratio | rs74603314 | 0.231 | 0.049 | 0.000 | -0.073 | 0.047 | 0.122 |
| order.MollicutesRF9.id.11579 | Adenosine 5'-diphosphate (ADP) to ornithine ratio | rs76373661 | 0.091 | 0.020 | 0.000 | -0.059 | 0.031 | 0.054 |
| order.MollicutesRF9.id.11579 | Adenosine 5'-diphosphate (ADP) to ornithine ratio | rs7706512 | -0.066 | 0.014 | 0.000 | -0.002 | 0.021 | 0.924 |
| order.MollicutesRF9.id.11579 | Adenosine 5'-diphosphate (ADP) to ornithine ratio | rs7801843 | -0.087 | 0.019 | 0.000 | -0.013 | 0.030 | 0.661 |
| order.MollicutesRF9.id.11579 | Adenosine 5'-diphosphate (ADP) to ornithine ratio | rs949341 | -0.066 | 0.015 | 0.000 | -0.026 | 0.022 | 0.247 |
| order.MollicutesRF9.id.11579 | Fructose to maltose ratio | rs10071529 | -0.125 | 0.028 | 0.000 | 0.005 | 0.034 | 0.873 |
| order.MollicutesRF9.id.11579 | Fructose to maltose ratio | rs114184530 | -0.155 | 0.034 | 0.000 | -0.004 | 0.042 | 0.930 |
| order.MollicutesRF9.id.11579 | Fructose to maltose ratio | rs11779863 | -0.077 | 0.017 | 0.000 | -0.019 | 0.021 | 0.359 |
| order.MollicutesRF9.id.11579 | Fructose to maltose ratio | rs13100746 | 0.064 | 0.014 | 0.000 | 0.020 | 0.018 | 0.264 |
| order.MollicutesRF9.id.11579 | Fructose to maltose ratio | rs17235252 | -0.122 | 0.026 | 0.000 | -0.005 | 0.030 | 0.875 |
| order.MollicutesRF9.id.11579 | Fructose to maltose ratio | rs3932485 | 0.063 | 0.014 | 0.000 | 0.024 | 0.018 | 0.176 |
| order.MollicutesRF9.id.11579 | Fructose to maltose ratio | rs515984 | 0.088 | 0.019 | 0.000 | 0.043 | 0.024 | 0.079 |
| order.MollicutesRF9.id.11579 | Fructose to maltose ratio | rs62188991 | -0.111 | 0.024 | 0.000 | -0.022 | 0.033 | 0.494 |
| order.MollicutesRF9.id.11579 | Fructose to maltose ratio | rs638542 | 0.071 | 0.016 | 0.000 | -0.010 | 0.020 | 0.630 |
| order.MollicutesRF9.id.11579 | Fructose to maltose ratio | rs739151 | -0.065 | 0.014 | 0.000 | 0.004 | 0.018 | 0.834 |
| order.MollicutesRF9.id.11579 | Fructose to maltose ratio | rs74603314 | 0.231 | 0.049 | 0.000 | 0.077 | 0.040 | 0.052 |
| order.MollicutesRF9.id.11579 | Fructose to maltose ratio | rs76373661 | 0.091 | 0.020 | 0.000 | 0.013 | 0.026 | 0.619 |
| order.MollicutesRF9.id.11579 | Fructose to maltose ratio | rs7706512 | -0.066 | 0.014 | 0.000 | -0.003 | 0.018 | 0.851 |
| order.MollicutesRF9.id.11579 | Fructose to maltose ratio | rs7801843 | -0.087 | 0.019 | 0.000 | -0.015 | 0.025 | 0.562 |
| order.MollicutesRF9.id.11579 | Fructose to maltose ratio | rs949341 | -0.066 | 0.015 | 0.000 | 0.010 | 0.019 | 0.586 |
| order.MollicutesRF9.id.11579 | Mannitolsorbitol | rs10071529 | -0.125 | 0.028 | 0.000 | -0.026 | 0.030 | 0.391 |
| order.MollicutesRF9.id.11579 | Mannitolsorbitol | rs114184530 | -0.155 | 0.034 | 0.000 | -0.016 | 0.036 | 0.661 |
| order.MollicutesRF9.id.11579 | Mannitolsorbitol | rs11779863 | -0.077 | 0.017 | 0.000 | -0.041 | 0.018 | 0.026 |
| order.MollicutesRF9.id.11579 | Mannitolsorbitol | rs13100746 | 0.064 | 0.014 | 0.000 | 0.003 | 0.016 | 0.866 |
| order.MollicutesRF9.id.11579 | Mannitolsorbitol | rs17235252 | -0.122 | 0.026 | 0.000 | -0.022 | 0.026 | 0.407 |
| order.MollicutesRF9.id.11579 | Mannitolsorbitol | rs3932485 | 0.063 | 0.014 | 0.000 | 0.005 | 0.016 | 0.762 |
| order.MollicutesRF9.id.11579 | Mannitolsorbitol | rs515984 | 0.088 | 0.019 | 0.000 | 0.016 | 0.021 | 0.457 |
| order.MollicutesRF9.id.11579 | Mannitolsorbitol | rs62188991 | -0.111 | 0.024 | 0.000 | 0.026 | 0.028 | 0.351 |
| order.MollicutesRF9.id.11579 | Mannitolsorbitol | rs638542 | 0.071 | 0.016 | 0.000 | -0.004 | 0.017 | 0.827 |
| order.MollicutesRF9.id.11579 | Mannitolsorbitol | rs739151 | -0.065 | 0.014 | 0.000 | 0.010 | 0.015 | 0.508 |
| order.MollicutesRF9.id.11579 | Mannitolsorbitol | rs74603314 | 0.231 | 0.049 | 0.000 | 0.036 | 0.034 | 0.299 |
| order.MollicutesRF9.id.11579 | Mannitolsorbitol | rs76373661 | 0.091 | 0.020 | 0.000 | -0.005 | 0.023 | 0.831 |
| order.MollicutesRF9.id.11579 | Mannitolsorbitol | rs7706512 | -0.066 | 0.014 | 0.000 | -0.015 | 0.015 | 0.349 |
| order.MollicutesRF9.id.11579 | Mannitolsorbitol | rs7801843 | -0.087 | 0.019 | 0.000 | -0.036 | 0.022 | 0.102 |
| order.MollicutesRF9.id.11579 | Mannitolsorbitol | rs949341 | -0.066 | 0.015 | 0.000 | -0.003 | 0.016 | 0.863 |

**Table S12 Full result of MR estimates for the association between gut microbiota and metabolite.**

| **Exposure** | **Outcome** | **Method** | **nSNP** | **MR analysis** | | | |
| --- | --- | --- | --- | --- | --- | --- | --- |
|  |  |  |  | **Beta** | **SE** | **P** | **OR** |
| genus.Catenibacterium.id.2153 | Glycerol 3-phosphate levels | IVW | 5 | -0.123 | 0.061 | 0.043 | 0.884 |
| genus.Dialister.id.2183 | 21-hydroxypregnenolone disulfate l | IVW | 30 | 0.093 | 0.044 | 0.037 | 1.097 |
| genus.Dialister.id.2183 | Inosine to EDTA ratio | IVW | 30 | 0.176 | 0.071 | 0.013 | 1.192 |
| genus.Dialister.id.2183 | Mannose to glycerol ratio | IVW | 30 | -0.055 | 0.041 | 0.177 | 0.946 |
| genus.Dialister.id.2183 | 1-1-enyl-palmitoyl GPC (p-160) | IVW | 10 | 0.190 | 0.081 | 0.019 | 1.209 |
| genus.Oscillospira.id.2064 | Adenosine 5'-diphosphate (ADP) to fructose ratio | IVW | 10 | -0.210 | 0.101 | 0.037 | 0.810 |
| genus.Oscillospira.id.2064 | Gamma-glutamylthreonine | IVW | 9 | -0.188 | 0.080 | 0.019 | 0.829 |
| genus.Ruminiclostridium5.id.11355 | 1-(1-enyl-palmitoyl)-2-oleoyl-GPE (p-16:0/18:1) levels | IVW | 13 | 0.175 | 0.086 | 0.042 | 1.191 |
| order.MollicutesRF9.id.11579 | Adenosine 5'-diphosphate (ADP) to fructose ratio | IVW | 12 | -0.231 | 0.100 | 0.021 | 0.794 |
| order.MollicutesRF9.id.11579 | Mannose to mannitol to sorbitol ratio | IVW | 12 | -0.147 | 0.075 | 0.048 | 0.863 |
| order.MollicutesRF9.id.11579 | 2-hydroxystearate | IVW | 12 | -0.129 | 0.065 | 0.048 | 0.879 |
| order.MollicutesRF9.id.11579 | Acetylcarnitine (C2) to propionylcarnitine (C3) ratio | IVW | 12 | -0.173 | 0.063 | 0.006 | 0.841 |
| order.MollicutesRF9.id.11579 | Adenosine 5'-diphosphate (ADP) to ornithine ratio | IVW | 12 | -0.181 | 0.089 | 0.041 | 0.835 |
| order.MollicutesRF9.id.11579 | Fructose to maltose ratio | IVW | 12 | 0.177 | 0.075 | 0.018 | 1.194 |
| order.MollicutesRF9.id.11579 | Mannitolsorbitol | IVW | 12 | 0.156 | 0.065 | 0.017 | 1.168 |

**Figure S1. Effects of different SNPs on GD.**

MR effect size of GD results for each SNP alone, and IVW and MR Egger analysis results for the combined effect of all SNPs on PAH for each bacteria. IVW, inverse variance weighted; SNP, singlenucleotide polymorphism; MR, mendelian randomization; GD, Graves’disease.

**Figure S2. Scatter plot of forward MR analysis between gut microbiota and GD.**

Four two-sample MR methods were used to study the relationship between the relative abundance of intestinal bacteria and GD using IVW, MR-Egger, weighted median, and weighted mode. A total of 8 intestinal microorganisms were found to be associated with GD.

**Figure S3. Heterogeneity analysis of GD and IVW.**

Funnel plot of heterogeneity analysis of IVW and MR Egger data between gut microbiota and GD.

**Figure S4. Leave-one-out analysis.**

For the intestinal flora with P < 0.05 in the IVW method and passing the heterogeneity test and horizontal pleiotropy test, the relevant SNPs were removed one by one, and the combined effects of the remaining SNPs were calculated. IVW, inverse variance weighting; SNP, single nucleotide polymorphism.
